# The role of gender in early childcare practices in low- and middle-income countries: a systematic review and meta-analysis

**DOI:** 10.7189/jogh.16.04057

**Published:** 2026-02-20

**Authors:** Manzura Jumaniyazova, Eliana Chavarría-Pino, Friederike Suhr, Cecilia Michelle Argueta, Janina Isabel Steinert

**Affiliations:** 1 TUM School of Social Sciences and Technology, Technical University of Munich, Munich, Germany; 2 Health Organisation, Policy, and Economics, Centre for Primary Care and Health Services Research. The University of Manchester, Manchester, UK; 3 Institute of Occupational, Social and Environmental Medicine, University Medical Center of the Johannes Gutenberg University Mainz, Mainz, Germany; 4 Global Health Action, Atlanta, USA; 5 Munich Center for Health Economics and Policy (M-CHEP), Munich, Germany

## Abstract

**Background:**

Gender disparities in early childcare practices impede gender equality and create long-lasting barriers to girls’ health, well-being, and future opportunities. Through this systematic review and meta-analysis, we aimed to quantitatively synthesise evidence on gender disparities in low- and middle-income countries (LMICs) across the World Health Organization’s five components of nurturing care: breastfeeding, immunisation, prenatal check-ups, postnatal check-ups, and healthcare expenditure for children under five.

**Methods:**

We searched sixteen scientific databases, journals, and repositories in November 2021 and again in January-February 2024, for studies examining gender differences in early childcare practices in LMICs, covering breastfeeding, immunisation, prenatal, and postnatal check-ups, and healthcare expenditure for children under five. We set no restrictions on publication type or date, but with limitations to English-language studies with sample sizes over 30. We standardised effect estimates from individual studies into Hedges’ g effect sizes and meta-analysed them using robust variance estimation. We assessed the quality of the included studies using the Joanna Briggs Institute quality appraisal tool.

**Results:**

We identified 78 eligible studies covering 55 LMICs, with 52 studies and 231 effect sizes included in the meta-analysis. The pooled analysis showed gender discrimination against girls across outcomes (52 studies; Hedges’ g = −0.082; 95% confidence interval (CI) = −0.133, 0.030), particularly pronounced in breastfeeding (17 studies; Hedges’ g = −0.051; 95% CI = −0.089, −0.012) and immunisation (32 studies; Hedges’ g = −0.073; 95% CI = −0.13, −0.016). While we also observed significant differences in favour of boys in pre- and post-natal check-ups (four studies; Hedges’ g = −0.029; 95% CI = −0.058, −0.000), the results were less robust due to a limited number of studies. We found no gender differences in healthcare spending patterns (seven studies; Hedges’ g = −0.278; 95% CI = −0.641, 0.083). Our meta-regression highlighted significant associations between a country’s ranking on the Gender Inequality Index and effect sizes, indicating stronger health-related penalties for girls. Effect sizes did not significantly vary by regions and the quality of included studies.

**Conclusion:**

s Our findings emphasise significant gender disparities in early childcare practices and point to the need for more evidence on inequalities in healthcare access and expenditures. We simultaneously observed signs of a narrowing gender gap in recent years, suggesting gradual progress toward more equitable child health outcomes.

**Registration:**

PROSPERO: CRD42021286151.

Son preference, entrenched in many societies, significantly influences parental decisions on early childcare practices. Such cultural norms not only disadvantage girls in early childhood, but also reinforce societal structures that hinder gender equality in the long run. While a recent systematic review concluded that boys are more likely to be undernourished [1], a large body of scientific evidence highlights that, in countries and settings with a high son preference, girls have higher mortality rates [2], are less likely to be fully immunised [3], are weaned earlier [4], have lower educational attainment [5], have worse long-term economic prospects [6], and have poorer mental health as adults [7], which in turn has detrimental long-term consequences for women and their offspring [8].

Addressing gender disparities in early childcare practices is vital for breaking the cycle of intergenerational poverty and ill health and fostering gender-equitable societal development. The first 1000 days from conception is crucial for a child’s long-term physical and cognitive development; during the first days of life, a child’s brain connects millions of neurons per second at a rate never equalled later [9]. Yet, around 250 million children are expected not to reach optimal development due to inadequate nutrition and care [10]. Children who fail to reach their developmental potential due to gender-biased practices are less likely to enjoy good health, educational success, or equal employment opportunities [6,8,10], directly contradicting several of the United Nations’ Sustainable Development Goals (SDGs).

In synthesising evidence on early childcare practices, previous reviews have focused on the links between children’s sex and exclusive breastfeeding in Ethiopia [11], on gender discriminatory care-seeking practices in South Asia [12] and on the global parent-level barriers to child vaccination [13,14]. Yet, to the best of our knowledge, these reviews have only presented narrative syntheses and have not comprehensively assessed the extent of gender discrimination in early childcare practices across different geographic locations and outcomes.

To address this gap, we conduct a systematic review and meta-analysis to quantitatively synthesise the existing evidence on the role of gender in early childcare practices in low- and middle-income countries (LMICs). Drawing on the five components of nurturing care proposed by the World Health Organization (WHO), we specifically assess gender disparities in immediate parental health investments, including breastfeeding, immunisation, pre- and post-natal check-ups, and healthcare expenditure [15]. Since a comprehensive systematic review and meta-analysis on gender differences in growth and nutrition-related outcomes has already been conducted [1], we deliberately exclude long-term outcomes such as growth and nutrition from our analysis. We also applied robust variance estimation, an advanced meta-analysis technique, to provide a nuanced, solid understanding of how gender influences early childcare practices.

## METHODS

### Search strategy and selection criteria

We sought to identify reports and studies on any potential gender difference in early childcare practices, specifically, in breastfeeding, immunisation, pre- and post-natal check-ups, and healthcare expenditure in LMICs. We registered the protocol with PROSPERO (CRD42021286151) and reported our findings per the PRISMA guidelines [16].

We systematically searched the following databases and organisational repositories for both scientific and grey literature: PubMed, Web of Science, Cochrane Library, EconPapers, EBSCO, NBER Working papers, World Bank Open Knowledge Repository, United Nations Children’s Fund, WHO, OpenGrey, Emergency Nutrition Network, and ProQuest, from 16 November 2021 to 24 November 2021, and again from 24 January 2024 to 25 February 2024. We applied no restrictions on publication date or type; records were retrieved from the earliest available entries in each database up to the most recent search date (Table S4 in the **Online Supplementary Document**). We further hand-searched the three journals (BMJ Global Health, Journal of Global Health, and Lancet Global Health), conducted a structured search in Google Scholar using predefined search teams, and reviewed the references of retrieved systematic reviews and meta-analyses.

We included studies that relied on quantitative cross-sectional or longitudinal data and reported gender-segregated information for any of the following outcomes for children under five years old: any breastfeeding, breastfeeding duration, immunisation status (full immunisation or single vaccination doses), the number of pre- and post-natal check-ups, and child-focused healthcare expenditure (any direct household spending on healthcare services for the child, including visits, medications, and treatment). Eligible studies needed to be conducted in LMICs, defined according to the World Bank 2024 definition [17] (Table S3 in the **Online Supplementary Document**), and written in English. Following the Central Limit Theorem, we excluded studies with fewer than 30 observations to ensure stable sampling distributions and to improve the reliability of the pooled effect size estimates [18]. We also excluded qualitative research, literature reviews, and systematic reviews.

One author (MJ) ran the initial searches and later performed an updated search alongside another author (FS). The search results were imported into the Rayyan online screening tool (Rayyan, Cambridge, Massachusetts, USA). After deduplication, rotating reviewer pairs independently screened all the titles and abstracts against the eligibility criteria (Table S5 in the **Online Supplementary Document**). Conflicts were resolved through discussion and consensus or by contacting a third reviewer. Subsequently, one author (MJ) performed the full-text screening, contacting a co-author (JS) in case of any uncertainty.

### Quality appraisal

We modified the Joanna Briggs Institute critical appraisal tool for cross-sectional studies to assess the quality of the included studies [19]. Given that our review focused on gender differences, rather than exposure-outcome relationships, we omitted exposure-related items and added others assessing whether study aims, objectives, and limitations were reported. We thus assessed the quality of the included studies using nine criteria about sampling, study setting, methods, and limitations of the study, with each criteria a value of 1 (met), 0.5 (partially met/unclear), or 0 (not met), and the total score being divided by nine to produce a standardised quality score ranging from 0 (lowest quality) to 1 (highest quality) (Table S6 in the **Online Supplementary Document**). We piloted the tool in 10% of studies, with the remaining studies assessed by three authors (EC, FS, CA). Any differences were discussed and resolved through consensus or by contacting another author (JS).

### Data extraction

We extracted the following characteristics: author, title, publication type (journal article, working paper, preprint, or bulletin), year of publication, region, country, study design, data source, year of data collection, sample size, age, outcome (breastfeeding/breastfeeding duration, complete immunisation, number of vaccinations, immunisation status for specific vaccines, number of pre-/post-natal check-ups, and child-related healthcare expenditure), and the corresponding effect sizes (*e.g.* odds ratios, risk ratios, ordinary least squares coefficients, means) into a piloted data extraction form. For studies where multiple effect sizes for the same outcome were available, we kept the most statistically rigorous ones from the estimation model including the highest number of control variables.

### Standardisation of effect sizes

To provide pooled estimates across all studies, we converted the study-specific estimates, where possible, to a common metric of a standardised mean difference, Cohen’s d, and its variance *V_d_*. For studies reporting odds ratio, we converted them to Cohen’s d values using the following formula:



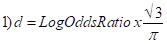





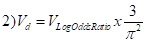



For studies that reported means for boys and girls, we calculated Cohen’s d as [20]:



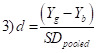





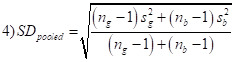



Here, *Y_g_* and *Y_b_* are mean values of the respective outcome variable, *s_g_*^2^ and *s_b_*^2^ stand for the standard deviation of these means, and *n_g_* and *n_b_* are sample sizes for girls and boys, respectively. For studies where we retrieved ordinary least square coefficients, we estimated the standardised mean difference as follows:



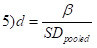





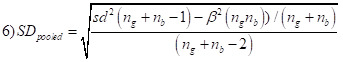



Here, *sd*^2^ is the standard deviation of the *β* coefficient.

Probit coefficients were first converted to log odds ratios using the following formula:



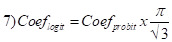



and then converted to Cohen’s d using equations 1) and 2).

To avoid overestimation due to small sample sizes, we applied Hedges’ small sample size correction and converted Cohen’s d to Hedges’ g using the following formula [21]:



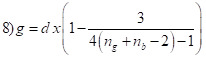



### Meta-analysis

The included studies may have had a statistical dependence due to correlated effect sizes, which arises if a single study reports multiple effect sizes or if a similar study population was used in several studies [22,23]. Since most of the studies relied on secondary data, some of them use identical datasets, such as the Demographic Health Survey and the National Family Health Survey. To adjust for dependency in included effect sizes, we applied a robust variance estimation (RVE) model, which takes into account the statistical dependence among the effect sizes, corrects the standard errors, and allows the inclusion of multiple effect sizes per study, regardless of their dependence structure [22,23]. Following the literature, we assumed correlated effect sizes and a rho of 0.8 for the observed effect sizes [24]. The estimated RVE model is defined as follows:



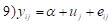



Here, *y_ij_* is the estimated effect size *i* in study *j* for the outcome of interest (breastfeeding, immunisation, prenatal and post-natal check-ups, or healthcare expenditure), *α* is the pooled average of the true effect, *u_j_* is a study-level random effect, *var*(*u_j_*) = *τ*^2^ is the between-study variance component, and *e_ij_* is an error term for *i*th effect size in the *j*th study. We performed the RVE for effect sizes pooled across all outcomes and separately by each outcome of interest. We present the between-study variance and proportion of observed differences due to heterogeneity rather than the sampling error using *τ*^2^ and *I*^2^ measures, respectively.

### Meta-regression

We ran meta-regressions to estimate the effect of selected study characteristics on standardised effect sizes using the following equation:



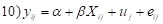



*X_ij_* is a vector of moderator variables, including geographic region, year of data collection, study quality, and the Gender Inequality Index (GII) score of the respective country. We included the GII in our meta-regression to test if countries with higher gender inequality ratings also find more pronounced gender differences in health outcomes between girls and boys. We ran meta-regressions by including each moderator variable separately and then by including all moderator variables together in one final estimation model.

We cleaned the data in Stata/MP, version 17.0 (StataCorp LLC, College Station, TX, USA) and performed the analysis in *R*, version 4.4.0 (R Foundation for Statistical Computing, Vienna, Austria).

### Publication bias

We assessed publication bias using the traditional methods of visual inspection of funnel plots and the formal Egger’s regression test, despite its sensitivity to heterogeneity and small sample sizes [25]. We also employed more recent methods, including visual inspection of DOI plots and the LFK index, for greater accuracy [26].

### Patient and public involvement

Patients were not involved in design, conduct, analysis, or reporting of this work.

## RESULTS

### Study selection

**Figure 1:**
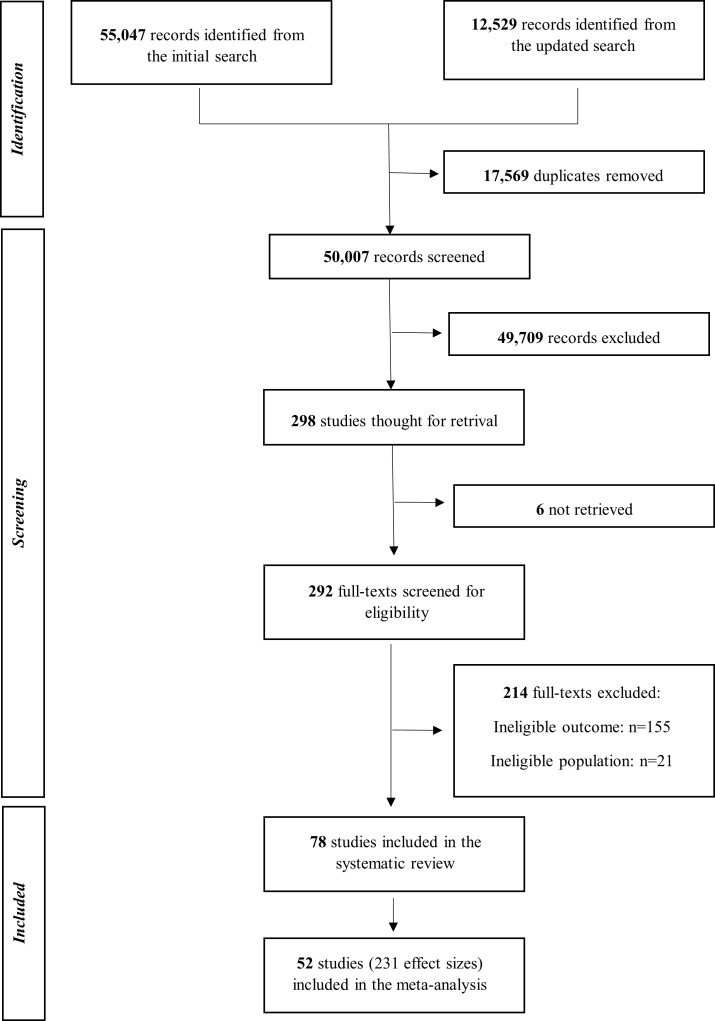
PRISMA flow diagram. Figures S1 and S2 in the **Online Supplementary Document** show the detailed number of studies retrieved from the original and updated searches from each of the data sources. n – number of studies.

We identified 67 576 records, of which 17 569 were duplicates, leaving 50 007 unique records for title and abstract screening **(****Figure 1****).** Following full-text screening of 298 articles, we included 78 studies in our systematic review [3,4,27–102] and 52 in the meta-analysis [3,4,28,30,32,34,37–40,43–46,48–54,57–63,65,69,71,73–75,77–79,81–86,88,90,92–94,99–102]. Twenty-six studies could not be included in the meta-analysis due to missing information on coefficients, standard deviations, or the number of observations. We contacted the corresponding authors of the studies with insufficient information, asking for additional data, and followed up with authors in case on non-response after two weeks. We dropped studies from the meta-analysis in case of non-response from the authors after the follow-up. Hazard ratios, risk reduction, risk differences, relative risk reduction, female-to-male ratios, Tobit estimates, accelerated failure time, and proportional hazard ratios were dropped from quantitative synthesis due to high heterogeneity in the nature of studies and/or due to insufficient data to calculate Cohen’s d and Hedges’ g values. Studies without sufficient data to calculate the standardised means scores were retained in the narrative synthesis. Specifically, we created a separate column in the table of included studies to summarise whether reported estimates suggested any health disadvantage for boys or for girls or reported null effects. Overall, 231 effect sizes were included in the analysis, with an average of 4.44 effect size estimates per study.

### Study characteristics

**Figure 2:**
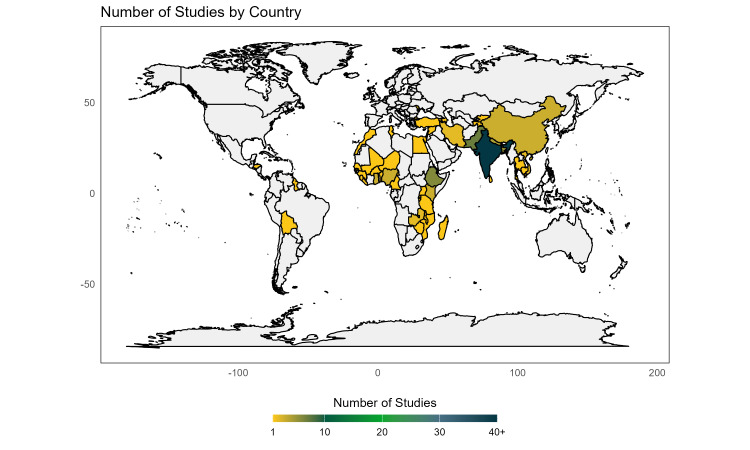
Geographical coverage of the included studies. *Estimations are based on 78 included studies identified in this review.

The 78 included studies covered 55 LMICs, with 54 (69%) focusing on South Asia, 11 (14%) on sub-Saharan Africa, 6 (8%) on the Middle East and North Africa, 4 (5%) on East Asia and Pacific, 2 (3%) on multiple countries and regions, and 1 (1%) on Europe and Central Asia. Forty-three (55%) studies focused on India, five (6%) on Ethiopia, and another five (6%) on Pakistan (**Figure 2**). Fifty-two (67%) studies focused on immunisation and 23 (30%) on breastfeeding, while only 7 (9%) assessed gender disparities in healthcare expenditure and 4 (5%) investigated pre- and post-natal check-ups. The sample size across studies ranged from 102 to over six million participants, with a total sample size of 16 936 585 across all studies included in the meta-analysis. The included studies were published between 1992 and 2024. 96% (n = 75) of included studies were journal articles, 3% (n = 2) were working papers or pre-prints, and 1% (n = 1) was a bulletin. In summary, 58% (n = 47) indicated that girls faced disadvantages, 40% (n = 32) showed no gender differences, 2% (n = 2) reported more favourable outcomes for girls (**Table 1**). The quality of included studies varied substantially between 22% (*i.e.* only 2 out of 9 criteria marked with highest quality) and 100%. This variation suggests significant differences in methodological quality, study conduct, and transparency in reporting across the included studies.

**Table 1:** Study characteristics*

Authors	Type	Year	Country	Region	Sample size	Breastfeeding	Immunisation	Pre- and post-natal check-ups	Healthcare expenditure	Summary for girls	Quality
Allan *et al*. [28]	A	2021	Kenya	Sub-Saharan Africa	3943		Y			No gender differences	81%
Antai [30]	A	2012	Nigeria	Sub-Saharan Africa	24 910		Y			Advantage	89%
Barcellos *et al*. [32]	A	2014	India	South Asia	110 00	Y	Y			Disadvantage	89%
Bharadvaj and Lakdawala [34]	A	2013	India, China, Bangladesh, Pakistan, Sri Lanka, Thailand, Ghana	Multiple	36 755			Y		Disadvantage	89%
Borooah [36]	A	2004	India	South Asia	4333		Y			Disadvantage	72%
Chakravarty [38]	A	2015	Egypt	Middle East and North Africa	48 304	Y				Disadvantage	67%
Chaudhry and Khan [39]	A	2020	India	South Asia	105 660	Y		Y		No gender differences in breastfeeding; disadvantage in postnatal check-ups.	67%
Chaudhuri [40]	A	2015	India	South Asia	777 705	Y	Y			No gender differences in breastfeeding; disadvantage in immunisation.	94%
Choi and Lee [41]	A	2006	India	South Asia	34 386		Y			Disadvantage	100%
Chowdhury *et al*. [42]	A	2003	Bangladesh	South Asia	3570		Y			Disadvantage	57%
Devasenapathy *et al*. [44]	A	2016	India	South Asia	1849		Y			Disadvantage	94%
Dixit *et al*. [45]	A	2020	India	South Asia	19 2151		Y			No gender differences	56%
Duraisamy and Duraisamy [46]	A	1995	India	South Asia	1272		Y			No gender differences	83%
Ganatra and Hirve [50]	B	1994	India	South Asia	456				Y	Disadvantage	72%
Geweniger and Abbas [52]	A	2020	Ethiopia	Sub-Saharan Africa	2004		Y			No gender differences	89%
Hajian-Tillaki [55]	A	2005	Iran	Middle East and North Africa	600		Y			No gender differences	72%
Jayachandran and Kuziemko [4]	A	2011	India	South Asia	110 000	Y				Disadvantage	94%
Mahmood *et al*. [64]	A	1994	Pakistan	South Asia	6558	Y	Y			Disadvantage	64%
Mishra *et al*. [65]	A	2004	India	South Asia	60 125	Y	Y			Disadvantage	78%
Nath and Goswami [67]	A	1997	India	South Asia	1650	Y				Disadvantage	89%
Nuruddin *et al*. [68]	A	2009	Pakistan	South Asia	3740				Y	No gender differences	83%
Obermeyer and Cardenas [69]	A	1997	Morrocco, Tunisia	Middle East and North Africa	6431	Y	Y			No gender differences in breastfeeding; disadvantage in immunisation	78%
Pande [3]	A	2003	India	South Asia	25 549		Y			Disadvantage	78%
Pande and Yazbeck [72]	A	2003	India	South Asia	89 777		Y			Disadvantage	57%
Pandey *et al*. [73]	A	2002	India	South Asia	530				Y	Disadvantage	72%
Patra [76]	W	2008	India	South Asia	304 162		Y			Disadvantage	64%
Prusty *et al*. [80]	A	2014	India	South Asia	NA		Y			Disadvantage	61%
Rajan and Morgan [81]	A	2018	India	South Asia	83 707	Y	Y			Disadvantage	72%
Rammohan *et al*. [82]	A	2014	India	South Asia	22 960		Y			Disadvantage	78%
Saini *et al*. [85]	A	2012	India	South Asia	161				Y	No gender differences	56%
Singh [91]	A	2011	India	South Asia	NA		Y			No gender differences	44%
Singh [92]	A	2012	India	South Asia	5587		Y			Disadvantage	89%
Singh [93]	A	2013	India	South Asia	30 601		Y			Disadvantage	83%
Singh [93]	A	2019	India	South Asia	4811	Y				Disadvantage	72%
Song *et al*. [95]	A	2020	Nepal	South Asia	1025		Y			No gender differences	61%
Srivastava and Navak [96]	A	1995	India	South Asia	NA		Y			Disadvantage	29%
Swenson *et al*. [97]	A	1993	Vietnam	East Asia and Pacific	4434	Y				No gender differences	50%
Vilms *et al*. [100]	A	2017	India	South Asia	11 570		Y	Y		Disadvantage	83%
Willis *et al*. [101]	A	2009	India	South Asia	255				Y	Disadvantage	83%
Altinkaynak *et al*. [29]	A	2004	Turkey	Europe and Central Asia	663		Y			No gender differences	64%
Arsenault *et al*. [31]	A	2017	Global	Multiple	63 233		Y			No gender differences	81%
Bhagyalaxmi *et al*. [33]	A	2007	India	South Asia	3073		Y			No gender differences	39%
Bhatia *et al*. [35]	A	2004	India	South Asia	796		Y			No gender differences	50%
Corsi *et al*. [43]	A	2009	India	South Asia	121 110		Y			Disadvantage	64%
Egondi *et al*. [47]	A	2015	Kenya	Sub-Saharan Africa	382		Y			Disadvantage	50%
Gaudin and Yazbeck [51]	A	2006	India	South Asia	20 157		Y			Disadvantage	64%
Hanifi *et al*. [56]	A	2018	Bangladesh	South Asia	4584		Y			Disadvantage	83%
Hu *et al*. [57]	A	2019	China	East Asia and Pacific	847		Y			No gender differences	83%
Jain and Bongraats [59]	A	2013	India	South Asia	1537	Y				Disadvantage	67%
Joe [60]	A	2015	India	South Asia	21 184		Y			Disadvantage	56%
Selvaraj *et al*. [88]	A	2017	India	South Asia	110	Y				Disadvantage	94%
Mugada *et al*. [66]	A	2017	India	South Asia	377		Y			Disadvantage	50%
Oster [71]	W	2006	India	South Asia	10 854		Y			Disadvantage	83%
Pillai and Conaway [78]	A	1992	Zambia	Sub-Saharan Africa	277		Y			No gender differences	67%
Pokhrel and Sauerborn [79]	A	2004	Nepal	South Asia	8112				Y	No gender differences	67%
Schoenbaum *et al*. [87]	A	1995	Gaza	Middle East and North Africa	4051	Y				No gender differences	72%
Shibre *et al*. [89]	A	2020	Ethiopia	Sub-Saharan Africa	7951		Y			No gender differences	67%
Budu *et al*. [37]	A	2022	Ghana	Sub-Saharan Africa	3650		Y			Disadvantage	50%
Fekadu *et al*. [48]	A	2024	Ethiopia	Sub-Saharan Africa	38 500		Y			No gender differences	72%
Fleddejohann and Channon [49]	A	2022	Nepal	South Asia	25648	Y				Disadvantage	89%
Ghosh *et al*. [53]	A	2022	India	South Asia	61431	Y				Disadvantage	83%
Iqbal *et al*. [58]	A	2023	Pakistan	South Asia	19 894			Y		Disadvantage	72%
Lai *et al*. [62]	A	2023	China	East Asia and Pacific	5294		Y			No gender differences	83%
Magalhães *et al*. [63]	A	2022	Ethiopia	Sub-Saharan Africa	102	Y				Advantage	78%
Saikia *et al*. [84]	A	2023	India	South Asia	43 291		Y			No gender differences	72%
Samuel *et al*. [86]	A	2022	Ethiopia	Sub-Saharan Africa	2036	Y				No gender differences	-
Siddiqi *et al*. [90]	A	2023	Pakistan	South Asia	6 235 305		Y			Disadvantage	86%
Taneja *et al*. [98]	A	2023	India	South Asia	NA		Y			Disadvantage	22%
Al-Akour *et al*. [27]	A	2014	Syria	Middle East and North Africa	334	Y				No gender differences	89%
Joshi *et al*. [61]	A	2014	Bangladesh	South Asia	121	Y				No gender differences	67%
Odusanya *et al*. [70]	A	2008	Nigeria	Sub-Saharan Africa	339		Y			No gender differences	67%
Parashar [74]	A	2005	India	South Asia	5623		Y			Disadvantage	100%
Partha and Bhattacharya [75]	A	2002	India	South Asia	11 748		Y			Disadvantage	67%
Phukan *et al*. [77]	A	2009	India	South Asia	616		Y			No gender differences	56%
Sahu *et al*. [83]	A	2010	India	South Asia	15 518		Y			Disadvantage	72%
Vafaee *et al*. [99]	A	2010	Iran	Middle East and North Africa	1450	Y				No gender differences	33%
Hafeez and Quintana-Domeque [54]	A	2018	Pakistan	South Asia	6955	Y				Disadvantage	100%
Yan and Ren [102]	A	2019	China	East Asia and Pacific	620				Y	No gender difference	100%

A – journal article, B – bulletin, W – working paper/preprint, NA – not available, Y – yes

*Summary statistics based on 78 eligible studies identified in this review. The summary of results is provided only with regard to gender and focuses only on the outcomes specified in this review (breastfeeding, immunisation, pre- and post-natal check-ups, and healthcare spending). When no rigorous statistical analyses were available, the summary was based on descriptive statistics. Please see the articles for additional results on other outcomes.

### Meta-analysis

**Figure 3:**
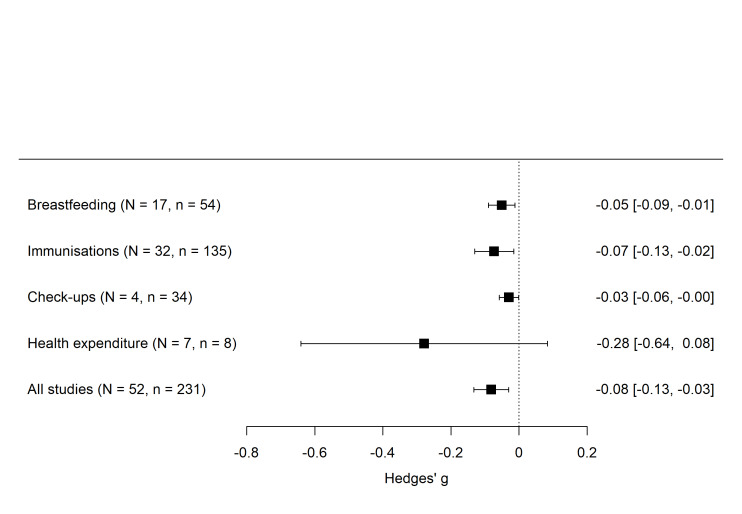
Forest plot of pooled effect sizes on the effect of gender on pooled health care outcomes and by each outcome type separately with 95% CI. Full results and forest plots of individual studies by outcome types are presented in Figures S3–6 and Tables S7–10 in the **Online Supplementary Document**. CI – confidence interval, N – number of studies, n – number of effect sizes in each group.

The majority of the 52 studies included in the meta-analysis concentrated on gender differences in immunisation (n = 32) and breastfeeding (n = 17), with only a few examining differences in pre-and post-natal check-ups (n =  4) and healthcare expenditures (n =  7). We found a negative and statistically significant overall effect of −0.082 (95% confidence interval (CI) = −0.133, −0.03; *P* = 0.002), indicating an overall health(care) disadvantage for girls (**Figure 3**). The *I*^2^ for the pooled estimates was high (98.75%), demonstrating high heterogeneity across studies that was not solely due to sampling error **(**Table S7 in the **Online Supplementary Document**). The analysis by outcome type showed more pronounced gender disparities for breastfeeding (Hedges’ g = −0.05; 95% CI = −0.089, −0.012; *P* = 0.013) and immunisation practices (Hedges’ g = −0.073; 95% CI = −0.13, −0.016; *P* = 0.015), with lower rates for girls compared to boys. Although the effect of pre- and post-natal check-ups was negative and significant (Hedges’ g = −0.029; 95% CI = −0.058, −0.000; *P* = 0.048), the associated degrees of freedom were 2.8 (**Figure 3**; Table S8 in the **Online Supplementary Document**). Results with less than four degrees of freedom should not be trusted as reliable [23], which is why we should interpret this finding with caution. Overall, since all pooled Hedges’ g values fell below 0.20, the observed gender differences represent small effect sizes. Nonetheless, these small effects translate into consistent disadvantages for girls, corresponding to approximately 9% lower breastfeeding, 12% lower immunisation, and 5.1% fewer pre- and post-natal check-ups compared to boys. We found no significant effect for health expenditure patterns when comparing boys and girls (Hedges’ g = −0.278; 95% CI = −0.641, 0.083; *P* = 0.109). These insignificant estimates may have stemmed from an absence of clear gender differences in healthcare spending or could have been related to the limited number of studies (n = 7) and effect sizes (n = 8) available for this outcome category. The *I*^2^ was over 70% across all sub-group specifications (Table S8 in the **Online Supplementary Document**). This high heterogeneity may have resulted from differences in outcome categories and from including different effect size measures. Note that *I*^2^ approaching 100% could have also been driven by a few studies where standard errors tend to approach zero due to large sample sizes [103].

Given that most of the studies in this review focused on India, we additionally ran a sensitivity analysis excluding studies with data from India (Table S10 in the **Online Supplementary Document**). The estimates for gender disparities across all outcomes were still negative, but no longer significant due to a loss of 31 studies and 160 effect sizes. Still, the consistently negative pattern of our results provided suggestive evidence that the observed negative pooled effect in the full sample of studies is not exclusively driven by the gender discrimination present in India.

### Meta-regression

We performed meta-regressions to explore the potential sources of the high heterogeneity (**Table 2**). Effect sizes did not vary significantly between geographical regions. However, there were less than four degrees of freedom, and the results must, therefore, be treated cautiously. The year of data collection was significantly and positively correlated with effect sizes, suggesting a narrowing gender gap over time. The study quality did not determine the direction and magnitude of effect sizes. We also ran the analysis using a dichotomous variable for study quality, dividing study into below and above median quality; the analysis yielded similarly insignificant results. As hypothesised, the GII exhibited a moderately significant negative correlation with effect sizes, indicating that higher levels of gender inequality in a country are associated with larger health-related penalties for girls. We did not find any significant effects in the full meta-regression that included all moderator variables simultaneously, except for the year of data collection, which indicated a decline in the gender gap over time. The *I*^2^ exceeded 98% in all specifications, implying that the high heterogeneity between included studies must have largely stemmed from factors not included in the meta-regression.

**Table 2:** RVE meta-regression results across all outcomes by region, publication type, year of data collection, quality and GII*

	Basic controls								Extended controls	
	**Coef (SE)**	**df**	**Coef (SE)**	**df**	**Coef (SE)**	**df**	**Coef (SE)**	**df**	**Coef**	**df**
**Region (omitted East Asia and Pacific**										
Middle East and North Africa	−0.065	3.98							−0.030	11.93
	0.070								0.294	
Sub-Saharan Africa	0.003	3.5							−0.385	12.44
	0.089								0.377	
South Asia	−0.115	2.33							−0.389	12.43
	0.073								0.375	
Multiple	−0.028	1.99							−0.238	11.22
	0.065								0.245	
**Year of data collection**			0.011†	23.3					0.022‡	7.31
			0.004						0.011	
**Quality**					−0.068	13.9			−0.080	20.94
					0.105				0.147	
**GII**							−0.565‡	6.75	1.357	8.78
							0.242		1.131	
***I* ^2^**	98.75%		98.57%		98.79%		98.7%		98.55%	

coef – coefficient, df – degrees of freedom, GII – Gender Inequality Index, SE – standard error

*Estimations with df less than four should be interpreted with caution. GII is measured across three dimensions: reproductive health, empowerment, and labour market outcome [104]. For studies conducted on multiple countries, we calculated the average GII for all included countries corresponding to the year of data collection. For countries where GII was unavailable for the specific year, we retrieved the score closest to that missing year. For papers that used multiple data sets over a period of time, we noted the data collection date as last year mentioned in the paper.

†*P* < 0.01.

‡*P* < 0.1.

### Publication bias

A visual inspection of the funnel plot suggested no asymmetry, corroborated by a *P*-value of 0.22 for the Egger’s regression test (Figure S7 in the **Online Supplementary Document**). In the absence of publication bias, the LFK value should lie between −1 and 1 [24]. Here, the estimated LFK value based on the DOI plot was −0.86 (Figure S8 in the **Online Supplementary Document**); hence, we conclude that our results were unlikely to be affected by publication bias.

## DISCUSSION

This systematic review explored gender differences in early childcare practices within LMICs. We identified 78 eligible studies covering 55 LMICs, of which 52 were included in the meta-analysis. We found evidence for significant gender discrimination against girls in parental health investments, including breastfeeding, immunisation, and pre- and post-natal check-ups. Our findings are alarming, given that adequate early childcare practices, as well as timely access to healthcare, are crucial determinants for child health and survival. Namely, breastfeeding is associated with a lower likelihood of contracting infectious diseases, higher intellectual abilities, and reduced risk of diabetes and being overweight [105]. Similarly, vaccinations in early life are estimated to prevent up to five million deaths annually [106]. A significant share of 2.6 million stillbirths and 2.7 million neonatal deaths could be averted with adequate and timely access to healthcare [107].

Very few reviews have focused on gender as a determinant of early childcare practices, with mixed conclusions. As opposed to our findings, qualitative evidence on child immunisation rarely identifies gender as a significant factor. For instance, a review of 25 qualitative studies reported that only one identified son preference as a parental barrier to child vaccination [14], while another review documented that only two out of 30 papers considered gender norms as a potential barrier [13]. In contrast, a review of quantitative studies on this topic, although limited in scope and focused primarily on India, pointed to a substantially higher vaccination coverage for boys than girls [108], in line with the pronounced gender differences in immunisation we observed here. Given that vaccinations are often provided free of charge in many countries, these gender-related disparities are likely driven by cultural norms and societal expectations around gender roles. Our findings also broaden the scope of existing reviews on the prevalence of pro-boy breastfeeding practices. For example, a review of studies conducted in Ethiopia found that newborn boys were 30% more likely to be exclusively breastfed than newborn girls in the first six months of their life [11]. A suggested underlying mechanism in the literature is fertility behaviour based on son preference, where mothers may use longer breastfeeding as a contraception method during boys’ infancy while shortening breastfeeding duration for infant girls to continue child-bearing in pursuit of having a boy [4].

Similarly, a previous systematic review on gender bias in healthcare access and utilisation documented wide gender disparities in favour of boys in hospital in-patient and out-patient attendance, duration from admission to death, and healthcare seeking behaviour, with the majority of studies conducted in Asia [109]. There is currently no systematic evidence on healthcare expenditure differences between boys and girls to compare our results to. Yet, a closer examination of included studies shows that healthcare expenditure patterns for boys and girls were not significantly different in China, Nepal, and Pakistan, and were slightly in favour of boys in India.

Gender-based differences also manifest in other dimensions of child development, extending beyond the outcomes addressed in this research. For example, despite a substantial progress towards achieving gender equality in education, girls (and women) still remain less educated than boys (and men) in many countries [110]. However, when focusing on nutritional status, a recent systematic review and meta-analysis found that boys are 14–29% more likely to be undernourished [1]. While this pattern is found more consistent across African countries, authors show that it diverges in other regions including East Africa, Central America, South and Southeast Asia, where the differences between boys and girls in undernutrition were either marginal or in favour of boys. These findings further highlight a gap in research explicitly examining the role of gender in healthcare investments and access.

This study has a few limitations that should be considered when interpreting its findings. First, as individual studies on pre- and post-natal care and healthcare expenditure are generally scarce, we are not able to include a larger number of related studies in this review, suggesting a cautious interpretation of observed gender differences in these dimensions. This limitation nevertheless underscores potential research avenues to explore gendered patterns in pre- and post-natal childcare as well as intra-household resource allocation among children. Second, a substantial share of the studies included in this review focused on India, which may limit the generalisability of our pooled findings to other contexts. This focus likely stems from the scientific interest in gender-related research in India, and more generally in South East Asia – especially following Amartya Sen’s seminal work on ‘missing women’ [111]. This region has long reported significant gender gaps in child development and mortality [2,3,112]. Nonetheless, other regions, such as East and Central Asia, which also exhibit strong son preference [113] but remain relatively understudied, need greater research efforts to uncover the patterns of gender inequalities and underlying contextual mechanisms. Third, a single reviewer conducted the full-text screening, which may have introduced a risk of selection bias or errors. However, this was mitigated by duplicate screening at earlier stages and by consulting JS in cases of uncertainty during full-text screening. Lastly, our language restriction is another shortcoming, as it may have excluded important studies written in languages other than English.

Despite these limitations, our systematic review and meta-analysis fill a critical gap in understanding the role of gender in early childcare practices, particularly in regions with a high prevalence of son preference. An important contribution of this study is its extensive scope, making it, to the best of our knowledge, the largest analysis of its kind. Additionally, we apply a rigorous quantitative approach to harmonise results based on diverse methodologies to systematically aggregate existing evidence. Another key strength of our review is the focus on child health ‘inputs’, as immediate parental investments in breastfeeding, child immunisation, and the use of healthcare services, rather than ‘outputs’, such as measures based on anthropometrics or mortality. This focus particularly underscores the urgency of continued efforts to understand the root causes of gender discrimination in early childcare practices, which ultimately contributes to closing gender gaps in adult life outcomes, and hence, more gender-equitable and prosperous societies. Policymakers should prioritise interventions that ensure equitable access to breastfeeding support, immunisation programs, and healthcare services for all children, regardless of gender.

## CONCLUSIONS

This systematic review and meta-analysis provide robust evidence that gender discrimination in early childcare practices remains a significant challenge in many LMICs. Across breastfeeding, immunisation, and pre- and post-natal healthcare, girls consistently receive fewer health investments than boys, despite the profound implications for child survival and long-term development. While the evidence base remains uneven across regions and topics, our findings underscore the urgent need for greater research attention in regions where strong son preference persists but empirical evidence is limited. Strengthening gender-sensitive health policies and ensuring equitable access to essential early-life services should be central priorities for governments and global health practitioners.

## Additional material


Online Supplementary Document
