# Supplementary material for: The role of gender in early childcare practices in low- and middle-income countries: a systematic review and meta-analysis
Source: J Glob Health. 2026 Feb 20;16:04057. doi: 10.7189/jogh.16.04057 (PMC12922561; doi:10.7189/jogh.16.04057)
Supplement: Online Supplementary Document [file jogh-16-04057-s001.pdf]

Supplement to: Jumaniyazova M, Chavarría-Pino E, Suhr F, Steinert JI. The role of gender in early childcare practices in low- and middle-income countries: a systematic review and meta-analysis. J Glob Health. 2026;16:04057.

SUPPLEMENTARY MATERIALS

Table S1: Abstract PRISMA checklist

Section and Topic	Item #	Checklist item	Reported (Yes/No)
**TITLE**			
Title	1	Identify the report as a systematic review.	p.1
**BACKGROUND**			
Objectives	2	Provide an explicit statement of the main objective(s) or question(s) the review addresses.	p.2
**METHODS**			
Eligibility criteria	3	Specify the inclusion and exclusion criteria for the review.	p.2
Information sources	4	Specify the information sources (e.g. databases, registers) used to identify studies and the date when each was last searched.	p.2
Risk of bias	5	Specify the methods used to assess risk of bias in the included studies.	p.2
Synthesis of results	6	Specify the methods used to present and synthesise results.	p.2
**RESULTS**			
Included studies	7	Give the total number of included studies and participants and summarise relevant characteristics of studies.	p.2
Synthesis of results	8	Present results for main outcomes, preferably indicating the number of included studies and participants for each. If meta-analysis was done, report the summary estimate and confidence/credible interval. If comparing groups, indicate the direction of the effect (i.e. which group is favoured).	p.2
**DISCUSSION**			
Limitations of evidence	9	Provide a brief summary of the limitations of the evidence included in the review (e.g. study risk of bias, inconsistency and imprecision).	p.2
Interpretation	10	Provide a general interpretation of the results and important implications.	p.2
**OTHER**			
Funding	11	Specify the primary source of funding for the review.	p.2
Registration	12	Provide the register name and registration number.	p.2

From: Page MJ, McKenzie JE, Bossuyt PM, Boutron I, Hoffmann TC, Mulrow CD, et al. The PRISMA 2020 statement: an updated guideline for reporting systematic reviews. BMJ 2021;372:n71. doi: 10.1136/bmj.n71

Table S2: *PRISMA Checklist*

Section and Topic	Item #	Checklist item	Location where item is reported
**TITLE**			
Title	1	Identify the report as a systematic review.	p.1
**ABSTRACT**			
Abstract	2	See the PRISMA 2020 for Abstracts checklist.	p.2
**INTRODUCTION**			
Rationale	3	Describe the rationale for the review in the context of existing knowledge.	p.3
Objectives	4	Provide an explicit statement of the objective(s) or question(s) the review addresses.	p.3
**METHODS**			
Eligibility criteria	5	Specify the inclusion and exclusion criteria for the review and how studies were grouped for the syntheses.	p.4, p.15 Supp. materials
Information sources	6	Specify all databases, registers, websites, organisations, reference lists and other sources searched or consulted to identify studies. Specify the date when each source was last searched or consulted.	p.4
Search strategy	7	Present the full search strategies for all databases, registers and websites, including any filters and limits used.	p.11-15 Supp. materials
Selection process	8	Specify the methods used to decide whether a study met the inclusion criteria of the review, including how many reviewers screened each record and each report retrieved, whether they worked independently, and if applicable, details of automation tools used in the process.	p.4
Data collection process	9	Specify the methods used to collect data from reports, including how many reviewers collected data from each report, whether they worked independently, any processes for obtaining or confirming data from study investigators, and if applicable, details of automation tools used in the process.	p.4-5
Data items	10a	List and define all outcomes for which data were sought. Specify whether all results that were compatible with each	p.5-6

Section and Topic	Item #	Checklist item	Location where item is reported
		outcome domain in each study were sought (e.g. for all measures, time points, analyses), and if not, the methods used to decide which results to collect.	
	10b	List and define all other variables for which data were sought (e.g. participant and intervention characteristics, funding sources). Describe any assumptions made about any missing or unclear information.	p.5, p.7
Study risk of bias assessment	11	Specify the methods used to assess risk of bias in the included studies, including details of the tool(s) used, how many reviewers assessed each study and whether they worked independently, and if applicable, details of automation tools used in the process.	p.4-5
Effect measures	12	Specify for each outcome the effect measure(s) (e.g. risk ratio, mean difference) used in the synthesis or presentation of results.	p.7-8
Synthesis methods	13a	Describe the processes used to decide which studies were eligible for each synthesis (e.g. tabulating the study intervention characteristics and comparing against the planned groups for each synthesis (item #5)).	p.5-6
	13b	Describe any methods required to prepare the data for presentation or synthesis, such as handling of missing summary statistics, or data conversions.	p.5-8
	13c	Describe any methods used to tabulate or visually display results of individual studies and syntheses.	p.8
	13d	Describe any methods used to synthesize results and provide a rationale for the choice(s). If meta-analysis was performed, describe the model(s), method(s) to identify the presence and extent of statistical heterogeneity, and software package(s) used.	p.6-7
	13e	Describe any methods used to explore possible causes of heterogeneity among study results (e.g. subgroup analysis, meta-regression).	p.7
	13f	Describe any sensitivity analyses conducted to assess robustness of the synthesized results.	p.18
Reporting bias assessment	14	Describe any methods used to assess risk of bias due to missing results in a synthesis (arising from reporting biases).	p.7-8
Certainty assessment	15	Describe any methods used to assess certainty (or confidence) in the body of evidence for an outcome.	n/a

Section and Topic	Item #	Checklist item	Location where item is reported
**RESULTS**			
Study selection	16a	Describe the results of the search and selection process, from the number of records identified in the search to the number of studies included in the review, ideally using a flow diagram.	p.9
	16b	Cite studies that might appear to meet the inclusion criteria, but which were excluded, and explain why they were excluded.	p. 7-8
Study characteristics	17	Cite each included study and present its characteristics.	p. 11-16
Risk of bias in studies	18	Present assessments of risk of bias for each included study.	p.11-16, p 26-29 in Supp. materials
Results of individual studies	19	For all outcomes, present, for each study: (a) summary statistics for each group (where appropriate) and (b) an effect estimate and its precision (e.g. confidence/credible interval), ideally using structured tables or plots.	p.18
Results of syntheses	20a	For each synthesis, briefly summarise the characteristics and risk of bias among contributing studies.	p.10
	20b	Present results of all statistical syntheses conducted. If meta-analysis was done, present for each the summary estimate and its precision (e.g. confidence/credible interval) and measures of statistical heterogeneity. If comparing groups, describe the direction of the effect.	p.18-20
	20c	Present results of all investigations of possible causes of heterogeneity among study results.	p.20
	20d	Present results of all sensitivity analyses conducted to assess the robustness of the synthesized results.	p.18
Reporting biases	21	Present assessments of risk of bias due to missing results (arising from reporting biases) for each synthesis assessed.	p.19
Certainty of evidence	22	Present assessments of certainty (or confidence) in the body of evidence for each outcome assessed.	n/a

Section and Topic	Item #	Checklist item	Location where item is reported
**DISCUSSION**			
Discussion	23a	Provide a general interpretation of the results in the context of other evidence.	p.21
	23b	Discuss any limitations of the evidence included in the review.	p.22
	23c	Discuss any limitations of the review processes used.	p.22
	23d	Discuss implications of the results for practice, policy, and future research.	p.22-23
**OTHER INFORMATION**			
Registration and protocol	24a	Provide registration information for the review, including register name and registration number, or state that the review was not registered.	p.4
	24b	Indicate where the review protocol can be accessed, or state that a protocol was not prepared.	p.4
	24c	Describe and explain any amendments to information provided at registration or in the protocol.	Supp. Materials p.15
Support	25	Describe sources of financial or non-financial support for the review, and the role of the funders or sponsors in the review.	p.23
Competing interests	26	Declare any competing interests of review authors.	p.23
Availability of data, code and other materials	27	Report which of the following are publicly available and where they can be found: template data collection forms; data extracted from included studies; data used for all analyses; analytic code; any other materials used in the review.	p.23

From: Page MJ, McKenzie JE, Bossuyt PM, Boutron I, Hoffmann TC, Mulrow CD, et al. The PRISMA 2020 statement: an updated guideline for reporting systematic reviews. BMJ 2021;372:n71. doi: 10.1136/bmj.n71. This work is licensed under CC BY 4.0. To view a copy of this license, visit https://creativecommons.org/licenses/by/4.0/

Table S3: World Bank Analytical Classifications (presented in World Development Indicators)

GNI per capita in US$ (Atlas methodology)

Bank's fiscal year:		**FY24**
Data for calendar year :		**2022**
Low income (L)		<= 1,145
Lower middle income (LM)		1,146 - 4,515
Upper middle income (UM)		4,516 - 14,005
High income (H)		> 14,005
AFG	Afghanistan	L
ALB	Albania	UM
DZA	Algeria	LM
AGO	Angola	LM
ARG	Argentina	UM
**ARM**	**Armenia**	**UM**
**AZE**	**Azerbaijan**	**UM**
**BGD**	**Bangladesh**	**LM**
BLR	Belarus	UM
BLZ	Belize	UM
**BEN**	**Benin**	**LM**
BTN	Bhutan	LM
**BOL**	**Bolivia**	**LM**
BIH	Bosnia and Herzogovina	UM
BWA	Botswana	UM
BRA	Brasil	UM
BGR	Bulgaria	UM
**BFA**	**Burkina Faso**	**L**
**BDI**	**Burundi**	**L**
CPV	Cabo Verde	LM
**KHM**	**Cambodia**	**LM**
**CMR**	**Cameroon**	**LM**
CAF	Central African Republic	L
**CHN**	**China**	**UM**

COL	Colombia	UM
TCD	Chad	L
**COM**	**Comoros**	**LM**
**COD**	**Congo, Dem. Rep.**	**L**
**COG**	**Congo, Rep.**	**LM**
CRI	Costa Rica	UM
**CIV**	**Côte d'Ivoire**	**LM**
CUB	Cuba	UM
DJI	Djibouti	LM
DMA	Dominica	UM
DOM	Dominican Republic	UM
ECU	Ecuador	UM
**EGY**	**Egypt, Arab Rep.**	**LM**
SLV	El Salvador	UM
GNQ	Equatorial Guinea	UM
ERI	Eritrea	L
SWZ	Eswatini	LM
**ETH**	**Ethiopia**	**L**
FJI	Fiji	UM
GAB	Gabon	UM
**GMB**	**Gambia, The**	**L**
**GHA**	**Ghana**	**LM**
GEO	Georgia	UM
GRD	Grenada	UM
GTM	Guatemala	UM
**GIN**	**Guinea**	**LM**
GNB	Guinea-Bissau	L
**HTI**	**Haiti**	**LM**
**HND**	**Honduras**	**LM**
**IND**	**India**	**LM**
**IDN**	**Indonesia**	**UM**
**IRN**	**Iran, Islamic Republic**	**LM**
IRQ	Iraq	UM
JAM	Jamaica	UM

JOR	Jordan	LM
KZH	Kazakhstan	UM
**KEN**	**Kenya**	**LM**
KIR	Kiribati	LM
XKX	Kosovo	UM
PRK	Korea, Dem. Rep.	L
**KGZ**	**Kyrgyz Republic**	**LM**
LAO	Lao PDR	LM
LBN	Lebanon	LM
**LSO**	**Lesotho**	**LM**
**LBR**	**Liberia**	**L**
LBY	Libya	UM
**MDG**	**Madagascar**	**L**
**MWI**	**Malawi**	**L**
MYS	Malaysia	UM
MDV	Maldives	UM
**MLI**	**Mali**	**L**
MHL	Marshall Islands	UM
MRT	Mauritania	LM
MUS	Mauritius	UM
MEX	Mexico	UM
FSM	Micronesia, Fed. Sts.	LM
**MOL**	**Moldova**	**UM**
MNE	Montenegro	UM
**MAR**	**Morocco**	**LM**
**MOZ**	**Mozambique**	**L**
MMR	Myanmar	LM
**NAM**	**Namibia**	**UM**
**NPL**	**Nepal**	**LM**
NIC	Nicaragua	LM
**NER**	**Niger**	**L**
**NGA**	**Nigeria**	**LM**
MKD	North Macedonia	UM
**PAK**	**Pakistan**	**LM**

PLW	Palau	UM
PNG	Papua New Guinea	LM
PRY	Paraguay	UM
PER	Peru	UM
PHL	Philippines	LM
RUS	Russian Federation	UM
**RWA**	**Rwanda**	**L**
WSM	Samoa	LM
**STP**	**São Tomé and Príncipe**	**LM**
**SEN**	**Senegal**	**LM**
SRB	Serbia	UM
**SLE**	**Sierra Leone**	**L**
SLB	Solomon Islands	LM
SOM	Somalia, Fed. Rep.	L
ZAF	South Africa	UM
SSD	South Sudan	L
**LKA**	**Sri Lanka**	**LM**
LCA	St. Lucia	UM
VCT	St. Vincent and the Grenadines	UM
SDN	Sudan	L
SUR	Suriname	UM
**SYR**	**Syrian Arab Republic**	**L**
**TJK**	**Tajikistan**	**LM**
**TZA**	**Tanzania**	**LM**
**THA**	**Thailand**	**UM**
**TLS**	**Timor-Leste**	**LM**
TGO	Togo	L
TON	Tonga	UM
**TUN**	**Tunisia**	**LM**
**TUR**	**Türkiye**	**UM**
TKM	Turkmenistan	UM
TUV	Tuvalu	UM
**UGA**	**Uganda**	**L**
UZB	Uzbekistan	LM

VUT	Vanuatu	LM
VEN	Venezuela, RB	
**VNM**	**Viet Nam**	**LM**
**PSE**	**West Bank and Gaza**	**UM**
YEM	Yemen, Rep.	L
**ZMB**	**Zambia**	**LM**
**ZWE**	**Zimbabwe**	**LM**

Note: Income classifications are set each year on July 1 for all World Bank member economies, and all other economies with populations of more than 30,000. These official analytical classifications are fixed during the World Bank's fiscal year (ending on June 30), thus economies remain in the categories in which they are classified irrespective of any revisions to their per capita income data. The historical classifications shown are as published on July 1 of each fiscal year.

Table S4: Search String

**PubMed**<br>(Initial search date: 17.11.2021<br>Updated search date: 24.01.2024)	(Breastfeed*[Title/Abstract] OR breast-feed*[Title/Abstract] OR breastfed*[Title/Abstract] OR wean*[Title/Abstract] OR Immuni?ation*[Title/Abstract] OR vaccin*[Title/Abstract] OR BCG*[Title/Abstract] OR (BCG vaccine)*[Title/Abstract] OR hepatisis*[Title/Abstract] OR (hepatisis B)[Title/Abstract] OR polio*[Title/Abstract] OR DTP*[Title/Abstract] OR (DTP-containing vaccine)[Title/Abstract] OR haemophilus*[Title/Abstract] OR (haemophilus influenzae)[Title/Abstract] OR pneumococcal*[Title/Abstract] OR rotavirus*[Title/Abstract] OR measle*[Title/Abstract] OR rubella*[Title/Abstract] OR HPV*[Title/Abstract] OR (Japanese encephalitis)[Title/Abstract] OR (yellow fever)[Title/Abstract] OR (tick-borne encephalitis)[Title/Abstract] OR typhoid*[Title/Abstract] OR cholera*[Title/Abstract] OR meningococcal*[Title/Abstract] OR (hepatisis A)[Title/Abstract] OR rabies*[Title/Abstract] OR dengue*[Title/Abstract] OR (dengue CYD-TDV)[Title/Abstract] OR (pre-natal check*)[Title/Abstract] OR (prenatal check*)[Title/Abstract] OR (post-natal check*)[Title/Abstract] OR (postnatal check*)[Title/Abstract] OR Nutrition*[Title/Abstract] OR malnutrition*[Title/Abstract] OR malnourished*[Title/Abstract] OR undernutrition*[Title/Abstract] OR undernourished*[Title/Abstract] OR (food insecure*)[Title/Abstract] OR (food security*)[Title/Abstract] OR (food expenditure)[Title/Abstract] OR (micronutrient deficiency)[Title/Abstract] OR stunting*[Title/Abstract] OR wasting*[Title/Abstract] OR underweight*[Title/Abstract] OR overweight*[Title/Abstract] OR obese*[Title/Abstract] OR obesity*[Title/Abstract] OR (Health expenditure)[Title/Abstract] OR (health spending)[Title/Abstract] OR (health care)[Title/Abstract] OR (medical care)[Title/Abstract]) AND ((gender difference*)[Title/Abstract] OR (gender bias)[Title/Abstract] OR (son preference)[Title/Abstract] OR (sex disaggregate*)[Title/Abstract] OR (gender discrimination)[Title/Abstract] OR (gender inequality)[Title/Abstract] OR (gender inequity)[Title/Abstract] OR (girl* NEXT2 discrimination)[Title/Abstract] OR (girl* NEXT2 disadvantage)[Title/Abstract] OR (female NEXT2 disadvantage)[Title/Abstract])
**EconPapers**<br>(Initial search date: 1.11.2021<br>Updated search date: 30.01.2024)	Breastfeed* OR breast-feed* OR breastfed* OR wean* OR Immuni?ation* OR vaccin* OR BCG* OR 'BCG vaccine' OR hepatisis* OR 'hepatisis B' OR polio* OR DTP* OR 'DTP-containing vaccine' OR haemophilus* OR 'haemophilus influenzae' OR pneumococcal* OR rotavirus* OR measle* OR rubella* OR HPV* OR 'Japanese encephalitis' or 'yellow fever' OR 'tick-borne encephalitis' OR typhoid* OR cholera* OR meningococcal* OR 'hepatisis A' OR rabies* OR dengue* OR 'dengue CYD-TDV' OR pre-natal check*' OR 'prenatal check*' OR 'post-natal check*' OR 'postnatal check*' OR Nutrition* OR malnutrition* OR malnourished* OR undernutrition* OR undernourished* OR 'food insecure*' OR 'food security*' OR 'food expenditure' OR 'micronutrient deficiency' OR stunting* OR wasting*

	or underweight* OR overweight* OR obese* OR obesity* OR Health expenditure' OR 'health spending' OR 'health care' OR 'medical care' AND Boy* OR girl* OR male* OR female* OR gender* OR sex* OR 'gender difference*' OR 'gender bias' OR 'son preference' OR 'sex disaggregate*'
**Cochrane library** (Initial search date: 16.11.2021 Updated search date: 24.01.2024)	Breastfeed* OR breast-feed* OR breastfed* OR wean* OR Immuni?ation* OR vaccin* OR BCG* OR (BCG vaccine)* OR hepatisis* OR (hepatisis B) OR polio* OR DTP* OR (DTP-containing vaccine) OR haemophilus* OR (haemophilus influenzae) OR pneumococcal* OR rotavirus* OR measle* OR rubella* OR HPV* OR (Japanese encephalitis) or (yellow fever) OR (tick-borne encephalitis) OR typhoid* OR cholera* OR meningococcal* OR (hepatisis A) OR rabies* OR dengue* OR (dengue CYD-TDV) OR (pre-natal check*) OR (prenatal check*) OR (post-natal check*) OR (postnatal check*) OR (Health expenditure) OR (health spending) OR (health care) OR (medical care) in Title Abstract Keyword AND Boy* OR girl* OR male* OR female* OR gender* OR sex* OR (gender difference*) OR (gender bias) OR (son preference) OR (sex disaggregate*) in Title Abstract Keyword - with Cochrane Library publication date Between Nov 2021 and Jan 2024, in Cochrane Reviews, Cochrane Protocols, Clinical Answers, Editorials, Special Collections (Word variations have been searched)
**Journal of Global Health** (Initial search date: 19.11.2021 Updated search date: 07.02.2024)	Breastfeed OR immunization OR prenatal care OR postnatal care OR (child health care)
**EBSCO** (Initial search date: 19.11.2021 Updated search date: 15.02.2024)	Breastfeed OR child immunization OR child vaccination OR prenatal OR post natal OR postnatal OR child health care
**Web of Science** (Initial search date: 17.11.2021 Updated search date: 07.02.2024)	(AB=((Health expenditure) OR (health spending) OR (health care) OR (medical care))) AND AB=((gender difference*) OR (gender bias) OR (son preference) OR (sex disaggregate*) OR (gender discrimination) OR (gender inequality) OR (gender inequity) OR (girl* NEXT2 discrimination) OR (girl* NEXT2 disadvantage) OR (female NEXT2 disadvantage)); ((AB=(Immunization* OR vaccin* OR BCG OR (BCG vaccine) OR hepatitis OR (hepatitis B) OR polio OR DTP OR (DTP-containing vaccine) OR haemophilus OR (haemophilus influenzae) OR pneumococcal OR rotavirus OR measle OR rubella OR HPV* OR (Japanese encephalitis) or (yellow fever) OR (tick-borne encephalitis) OR typhoid OR cholera OR meningococcal OR (hepatitis A) OR rabies OR dengue OR (dengue CYD-TDV))) AND AB=((gender difference*) OR (gender bias) OR (son preference) OR (sex disaggregate*) OR (gender discrimination) OR (gender inequality)

	OR (gender inequity) OR (girl* NEXT2 discrimination) OR (girl* NEXT2 disadvantage) OR (female NEXT2 disadvantage))); (AB=(Breastfeed* OR breast-feed* OR breastfed* OR wean*)) AND AB=((gender difference*) OR (gender bias) OR (son preference) OR (sex disaggregate*) OR (gender discrimination) OR (gender inequality) OR (gender inequity) OR (girl* NEXT2 discrimination) OR (girl* NEXT2 disadvantage) OR (female NEXT2 disadvantage)) and English (Languages); ((AB=((pre-natal check*) OR (prenatal check*) OR (post-natal check*) OR (postnatal check*))) AND AB=((gender difference*) OR (gender bias) OR (son preference) OR (sex disaggregate*) OR (gender discrimination) OR (gender inequality) OR (gender inequity) OR (girl* NEXT2 discrimination) OR (girl* NEXT2 disadvantage) OR (female NEXT2 disadvantage)))
**World Bank**<br>(Initial search date: 24.11.2021)	breastfeeding AND (gender difference); child immunization AND gender difference; gender differences in prenatal care; gender differences in postnatal care; gender differences in child nutrition; gender differences in child health care
**BMJ**<br>(Initial search date: 19.11.2021<br>Updated search date: 24.01.2024)	for abstract or title """child immunization" OR "child vaccination"" AND ""gender difference*" OR "gender bias" OR "son preference""" (match any words); """prenatal check" OR "postnatal check"" AND "gender bias" OR "son preference" OR "gender inequality"""; for abstract or title ""Breastfeed* OR breast-feed* OR breastfed* OR wean*" AND ""gender difference*" OR "gender bias" OR "son preference""" (match any words); abstract or title ""child health care" AND "gender inequality"" (match all words)
**The Lancet**<br>(Initial search date: 17.11.2021<br>Updated search date: 24.01.2024)	Breastfeed* OR breast-feed* OR breastfed* OR wean* AND “gender difference*” OR “gender bias” OR “son preference” OR “sex disaggregate*” OR “gender discrimination” OR “gender inequality” OR “gender inequity” OR “girl* NEXT2 discrimination” OR “girl* NEXT2 disadvantage” OR “female NEXT2 disadvantage”; Immuni?ation* OR vaccin* OR BCG OR “BCG vaccine” OR hepatitis* OR “hepatitis B” OR polio OR DTP OR “DTP-containing vaccine” OR haemophilus OR “haemophilus influenzae” OR pneumococcal* OR rotavirus OR measle OR rubella OR HPV OR “Japanese encephalitis” or “yellow fever” OR “tick-borne encephalitis” OR typhoid OR cholera OR meningococcal OR “hepatitis A” OR rabies OR dengue OR “dengue CYD-TDV” AND “gender difference*” OR “gender bias” OR “son preference” OR “sex disaggregate*” OR “gender discrimination” OR “gender inequality” OR “gender inequity” OR “girl* NEXT2 discrimination” OR “girl* NEXT2 disadvantage” OR “female NEXT2 disadvantage”; “pre-natal check*” OR “prenatal check*” OR “post-natal check*” OR “postnatal check*” AND “gender difference*” OR “gender bias” OR “son preference” OR “sex disaggregate*” OR “gender discrimination” OR “gender inequality” OR “gender inequity” OR “girl* NEXT2 discrimination” OR “girl* NEXT2 disadvantage” OR “female NEXT2 disadvantage”; “Health expenditure” OR “health spending” OR “health care” OR “medical care” AND “gender difference*” OR “gender bias” OR “son preference” OR “sex disaggregate*” OR “gender discrimination” OR

	“gender inequality” OR “gender inequity” OR “girl* NEXT2 discrimination” OR “girl* NEXT2 disadvantage” OR “female NEXT2 disadvantage”
**NBER**<br>(Initial search date: 19.11.2021<br>Updated search date: 15.02.2024)	Breastfeeding; immunization; vaccination; prenatal; postnatal; child health care
**OKR**<br>(Initial search date: 21.11.2021<br>Updated search date: 30.01.2024)	Breastfeeding; immunization; vaccination; prenatal; postnatal; child health care
**UNICEF**<br>(Initial search date: 22.11.2021<br>Updated search date: 15.02.2024)	Breastfeeding; immunization; vaccination; prenatal; child health care
**WHO**<br>(Initial search date: 24.11.2021<br>Updated search date: 15.02.2024)	Breastfeeding; immunization; vaccination; prenatal; postnatal; child health care
**OpenGrey**<br>(Initial search date: 21.11.2021<br>Updated search date: 15.02.2024)	(Breastfeed* OR breast-feed* OR breastfed* OR wean*) AND gender; immunization AND gender; vaccination AND gender; prenatal care; postnatal care; child health care AND gender
**ENN**<br>(Initial search date: 21.11.2021<br>Updated search date: 15.02.2024)	(Breastfeed* OR breast-feed* OR breastfed* OR wean*) AND (gender bias); (child immunization) AND (gender bias); (child vaccination) AND (gender bias); (prenatal care) AND (gender bias); (postnatal care) AND (gender bias); (health care) AND (gender bias)
**ProQuest**<br>(Initial search date: 17.11.2021<br>Updated search date: 30.01.2024)	ab(Breastfeed* OR breast-feed* OR breastfed* OR wean*) AND ab("gender difference*" OR "gender bias" OR "son preference" OR "sex disaggregate*" OR "gender discrimination" OR "gender inequality" OR "gender inequity" OR "girl* NEXT2 discrimination" OR "girl* NEXT2 disadvantage" OR "female NEXT2 disadvantage"); ab(Immuni?ation* OR vaccin* OR BCG OR “BCG vaccine” OR hepatitis* OR “hepatitis B” OR polio OR DTP OR “DTP-containing vaccine” OR haemophilus OR “haemophilus influenzae” OR pneumococcal* OR rotavirus OR measle OR rubella OR HPV OR “Japanese encephalitis” or “yellow fever” OR “tick-borne encephalitis” OR typhoid OR cholera OR meningococcal OR “hepatitis A” OR rabies OR dengue OR “dengue CYD-TDV”) AND ab("gender difference*" OR "gender bias" OR "son preference" OR "sex disaggregate*" OR "gender discrimination" OR "gender inequality" OR "gender inequity"

OR "girl* NEXT2 discrimination" OR "girl* NEXT2 disadvantage" OR "female NEXT2 disadvantage"); ab("pre-natal check*" OR "prenatal check*" OR "post-natal check*" OR "postnatal check*") AND ab("gender difference*" OR "gender bias" OR "son preference" OR "sex disaggregate*" OR "gender discrimination" OR "gender inequality" OR "gender inequity" OR "girl* NEXT2 discrimination" OR "girl* NEXT2 disadvantage" OR "female NEXT2 disadvantage"); ab("Health expenditure" OR "health spending" OR "health care" OR "medical care") AND ab("gender difference*" OR "gender bias" OR "son preference" OR "sex disaggregate*" OR "gender discrimination" OR "gender inequality" OR "gender inequity" OR "girl* NEXT2 discrimination" OR "girl* NEXT2 disadvantage" OR "female NEXT2 disadvantage")

Note: We excluded one of the research questions about the nutrition of children under five due to publication of a systematic review on the topic.

Table S5: Inclusion and Exclusion Criteria

**Domain**	**Inclusion criteria**	**Exclusion criteria**
Population	Children 0-59 months old of both sexes, regardless of who reports the outcomes	Children outside of 0-59 months old age range
Outcomes	**Breastfeeding:**<br>Breastfeeding dummy and breastfeeding duration<br>**Immunisation:**<br>Full immunisation, individual vaccine immunisation status<br>**Prenatal and post-natal check-ups**<br>**Healthcare expenditure**	Breastfeeding cessation, zero-dose immunisation prevalence
Setting	Low- and middle-income countries	High-income countries
Methodology	Quantitative cross-sectional or longitudinal studies that reported outcomes of interest and correlation/means/ prevalence for boys and girls along with standard deviations and number of observations by gender of the child under five years old and had a sample size of more than 30 observations	Qualitative studies, literature reviews, systematic reviews, and meta-analysis
Timeframe	No restrictions on the publication date	
Publication type	Journal articles, working papers, preprints, bulletins, technical reports	Policy briefs, country reports, editorial notes, abstracts, conference presentations with insufficient information to identify eligibility criteria
Language	Studies in English	Studies published in all other languages

Note: The references list of literature reviews, systematic reviews, and meta-analyses were checked to identify additional eligible studies.

Table S6. JBI Critical Appraisal Checklist For Analytical Cross-Sectional Studies

Reviewer_______________________________________
Date__________________________________

Author_______________________________________Year_________ Record Number_________

	Yes	No	Unclear	Not applicable
1. Were the aims and objectives of the study clearly stated?	□	□	□	□
2. Were the criteria for inclusion in the sample clearly defined?	□	□	□	□
3. Were the study subjects described in detail?	□	□	□	□
4. Was the study setting described in detail?	□	□	□	□
5. Were the outcomes measured in a valid and reliable way?	□	□	□	□
6. Were the confounding factors identified?	□	□	□	□
7. Were strategies to deal with confounding factors stated?	□	□	□	□
8. Was appropriate statistical analysis used?	□	□	□	□
9. Were the limitations of the study discussed?	□	□	□	□

Overall appraisal: Include □ Exclude □ Seek further info □

Comments (Including reason for exclusion)

EXPLANATION OF ANALYTICAL CROSS-SECTIONAL STUDIES CRITICAL APPRAISAL

Analytical cross-sectional studies Critical Appraisal Tool

Answers: Yes, No, Unclear or Not Applicable (NA)

1. **Were the aims and objectives of the study clearly stated?**

   The authors should provide the aims and objectives of the study with sufficient detail.

2. **Were the criteria for inclusion in the sample clearly defined?**

   The authors should provide clear inclusion and exclusion criteria that they developed prior to the recruitment of the study participants. The inclusion/exclusion criteria should be specified (e.g., child's age, maternal characteristics, place of residence, etc.) with sufficient detail and all the necessary information critical to the study.

3. **Were the study subjects described in detail?**

   The study sample should be described in sufficient detail so that other researchers can determine if it is comparable to the population of interest to them. The authors should provide a clear description of the population from which the study participants were selected or recruited, including demographics, and time period.

4. **Was the study setting described in detail?**

   The study setting should be described in sufficient detail (e.g. location, income levels, etc.) so that other researchers can determine if it is comparable to the population of interest to them.

5. **Were confounding factors identified?**

   A high-quality study will identify the potential confounders and measure them (where possible). This is difficult for studies where behavioral, attitudinal, or lifestyle factors may impact the results.

6. **Were strategies to deal with confounding factors stated?**

   Strategies to deal with the effects of confounding factors may be dealt within the study design or in data analysis. By matching or stratifying the sampling of participants, the effects of confounding factors can be adjusted for. When dealing with adjustments in data analysis, assess the statistics used in the study. Most will be some form of multivariate regression analysis to account for the confounding factors measured.

7. **Were the outcomes measured in a valid and reliable way?**

   Read the methods section of the paper. If e.g. vaccination is assessed based on existing definitions or diagnostic criteria, then the answer to this question is likely to be yes. If vaccination is assessed using observer-reported, or self-reported scales, the risk of over- or under-reporting is increased, and objectivity is compromised. Importantly, determine if the measurement tools used were validated instruments as this has a significant impact on outcome assessment validity.

8. **Was appropriate statistical analysis used?**

As with any consideration of statistical analysis, consideration should be given to whether there was a more appropriate alternate statistical method that could have been used. The methods section should be detailed enough for reviewers to identify which analytical techniques were used (in particular, regression or stratification) and how specific confounders were measured.

For studies utilizing regression analysis, it is useful to identify if the study identified which variables were included and how they related to the outcome. If stratification was the analytical approach used, were the strata of analysis defined by the specified variables? Additionally, it is also important to assess the appropriateness of the analytical strategy in terms of the assumptions associated with the approach as differing methods of analysis are based on differing assumptions about the data and how it will respond.

9. Were the limitations of the study discussed?

The authors should discuss the potential limitations of the study and discuss the implications of these limitations on the results.

Figure S1. PRISMA flow diagram (initial search)

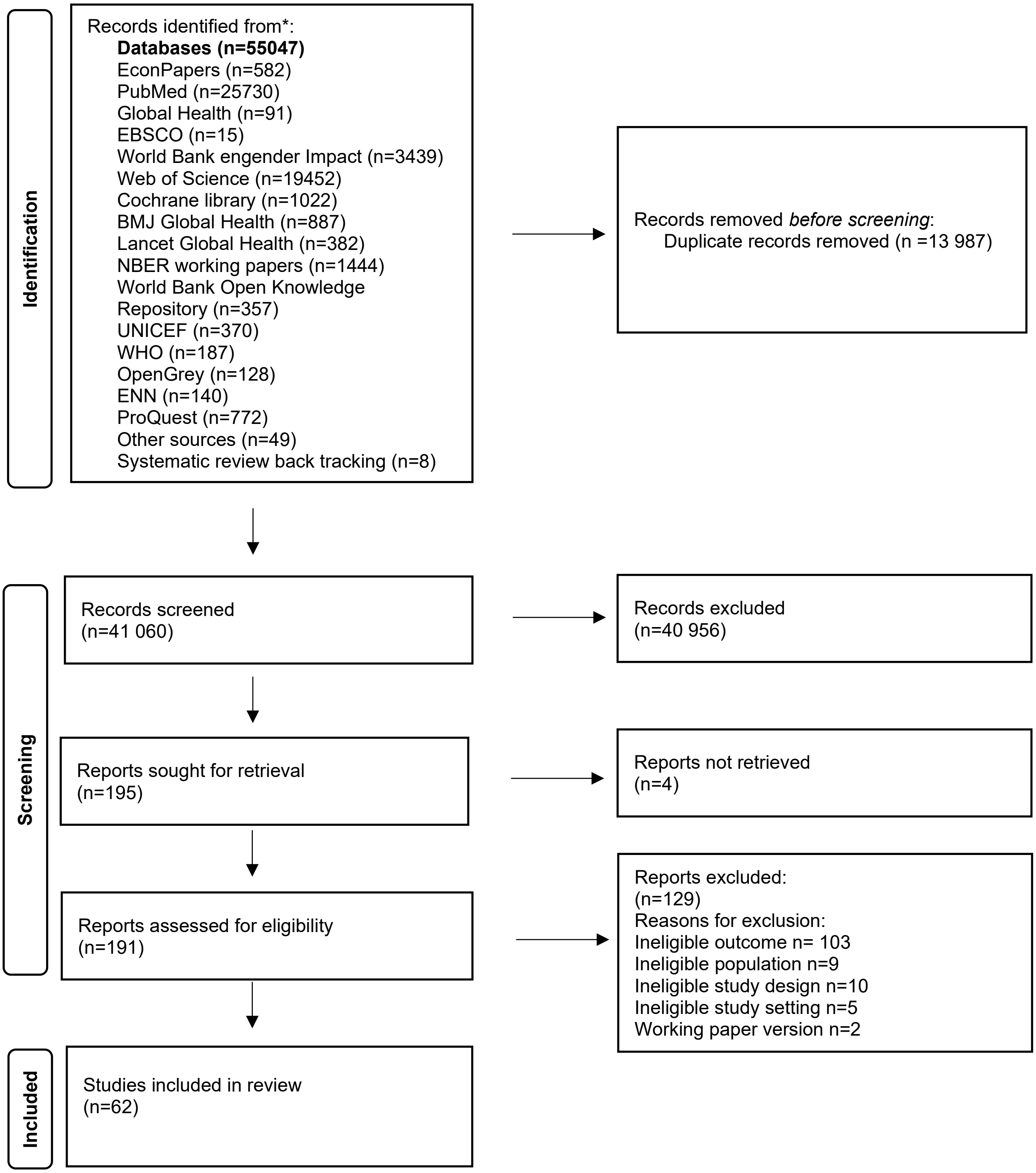

Figure S2. PRISMA flow diagram (updated search)

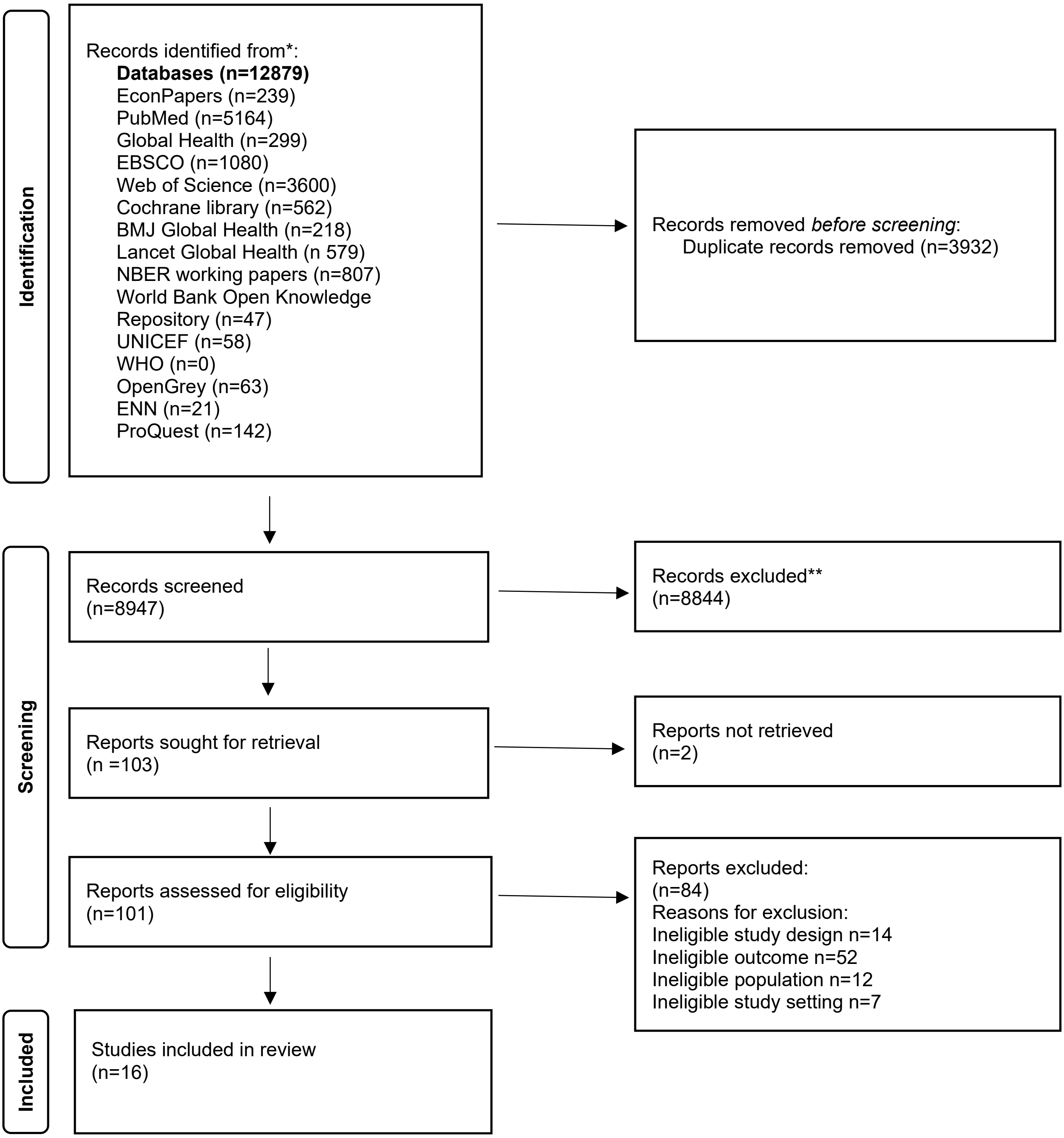

Table S7. Pooled effect sizes across all outcome types

	All outcomes
**Hedges' g**	-0082***
**SE**	0.026
**95% CI**	[-0.133, - 0.03]
**I^2 (consistency)**	98.75%
**τ^2 (heterogeneity)**	0.031
**Degree of freedom**	49.7
**N of studies**	52
**N of effect sizes**	231

Note: *** p<0.01, ** p<0.05, * p<0.1. Coefficients with a degree of freedom less than four should be interpreted cautiously.

Table S8. RVE estimation by each outcome type

	Breastfeeding	Immunisation	Pre- and Post-natal check-ups	Healthcare expenditure
**Hedges' g**	-0.051**	-0.073**	-0.029**	-0.278
**SE**	0.02	0.029	0.009	0.148
**95% CI**	[-0.089, -0.012]	[-0.13, -0.016]	[-0.058, -0.001]	[-0.641, 0.084]
**I^2 (consistency)**	95.74%	99.2%	71.88%	91.4%
**τ^2 (heterogeneity)**	0.007	0.04	0.002	0.126
**Degree of freedom**	13.8	30.8	2.78	5.97
**N of studies**	17	32	4	7
**N of effect sizes**	54	135	34	8

Note: *** p<0.01, ** p<0.05, * p<0.1. Coefficients with a degree of freedom less than four should be interpreted cautiously.

Table S9. RVE estimation by immunisation type

	Immunisation	Full immunisation	Individual vaccine
**Hedges' g**	-0.073**	-0.097**	-0.052**
**SE**	0.029	0.044	0.018
**95% CI**	[-0.13, -0.016]	[-0.188, -0.006]	[-0.092, -0.012]
**I^2 (consistency)**	99.2%	99.49	89.15
**τ^2 (heterogeneity)**	0.04	0.084	0.002
**Degree of freedom**	30.8	18	11.5
**N of studies**	32	20	14
**N of effect sizes**	135	65	70

Note: *** p<0.01, ** p<0.05, * p<0.1. Coefficients with a degree of freedom less than four should be interpreted cautiously.

Table S10. Sensitivity analysis excluding India

	All outcomes
**Hedges' g**	-0.022
**SE**	0.023
**95% CI**	[-0.069, 0.025]
**I^2 (consistency)**	94.31%
**τ^2 (heterogeneity)**	0.011
**Degree of freedom**	18.3
**N of studies**	21
**N of effect sizes**	71

Note: *** p<0.01, ** p<0.05, * p<0.1. Coefficients with a degree of freedom less than four should be interpreted cautiously.

Figure S3. Forest plot of individual studies in breastfeeding and corresponding pooled RVE model estimate.

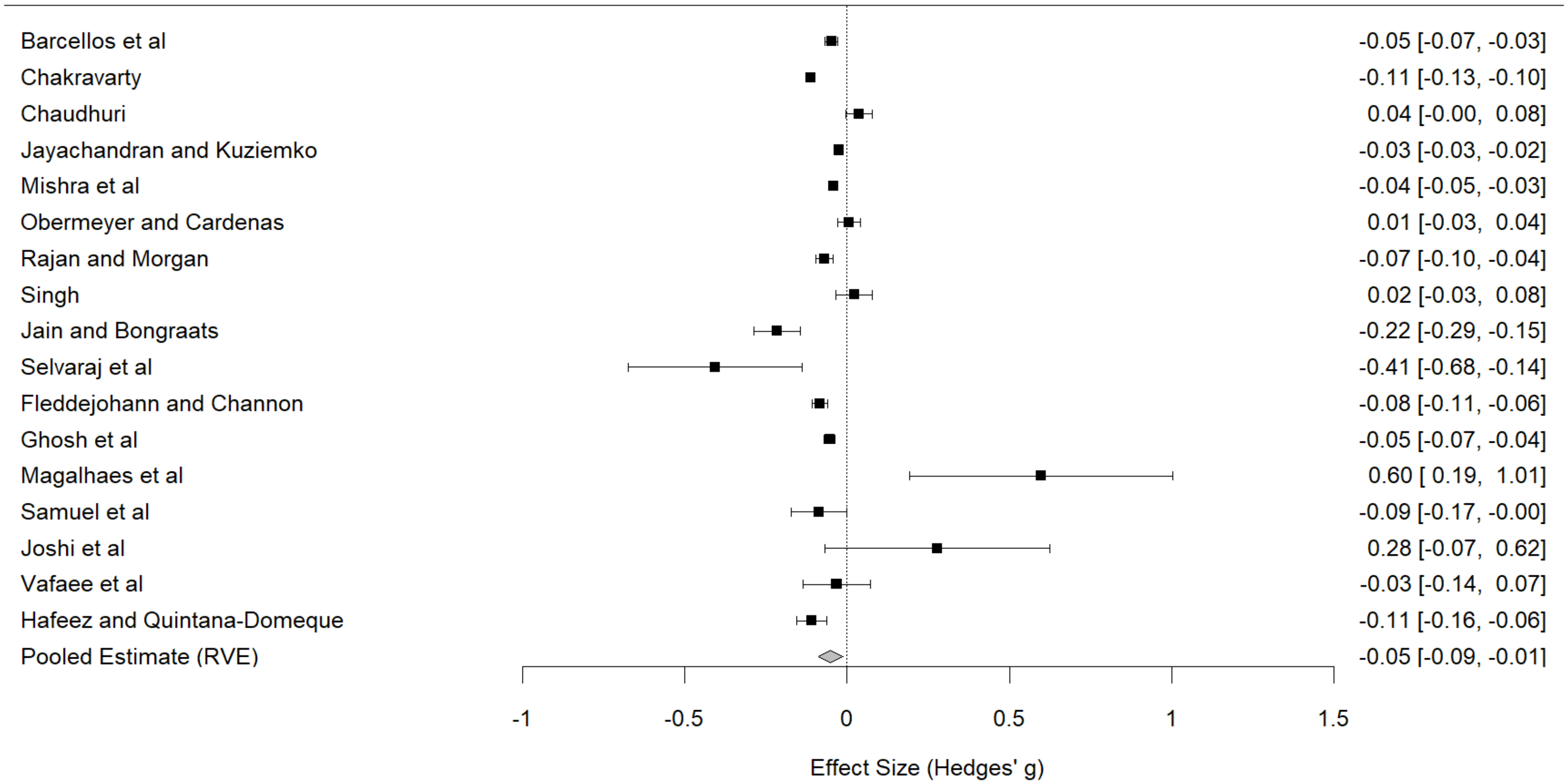

Figure S4. Forest plot of individual studies in immunisation and corresponding pooled RVE model estimate.

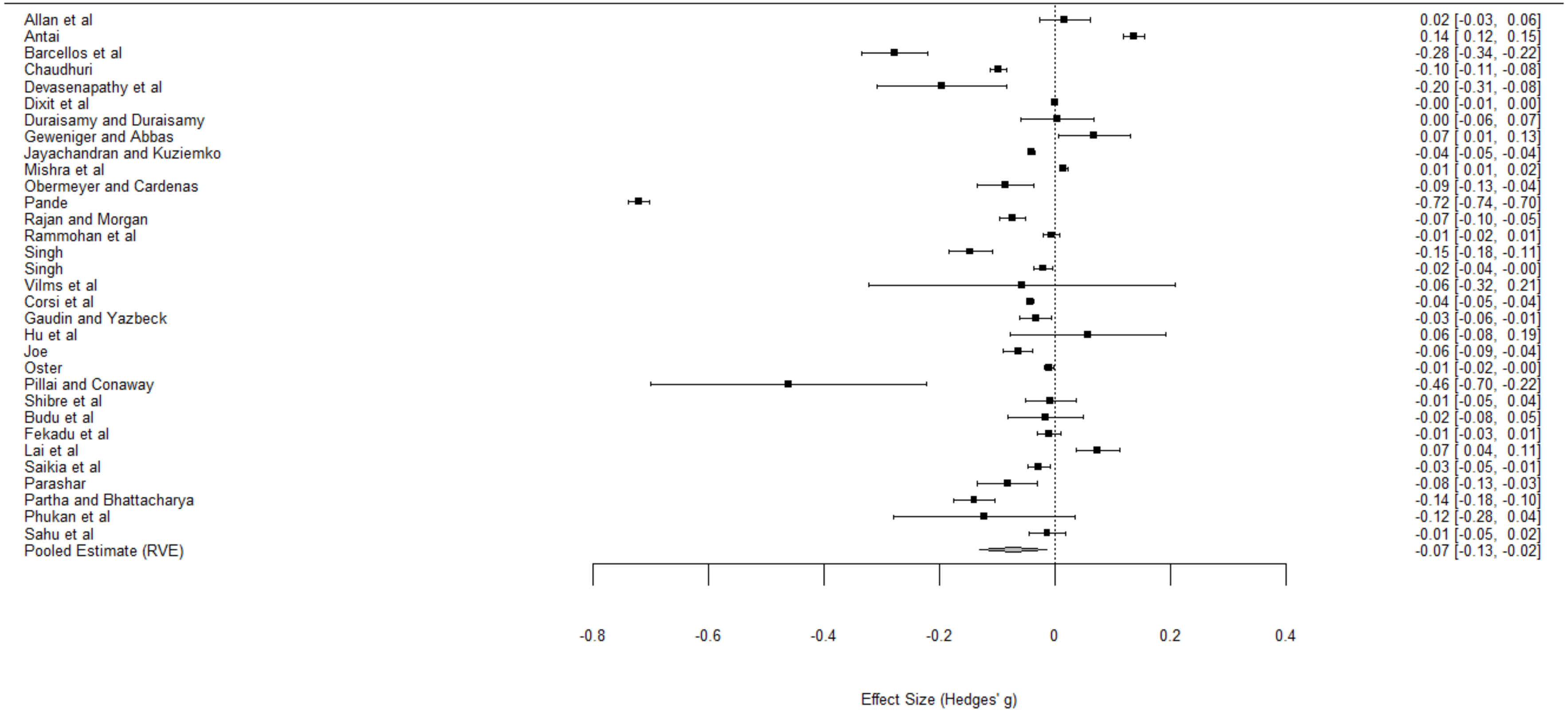

Figure S5. Forest plot of individual studies in pre- and post-natal check-ups and corresponding pooled RVE model.

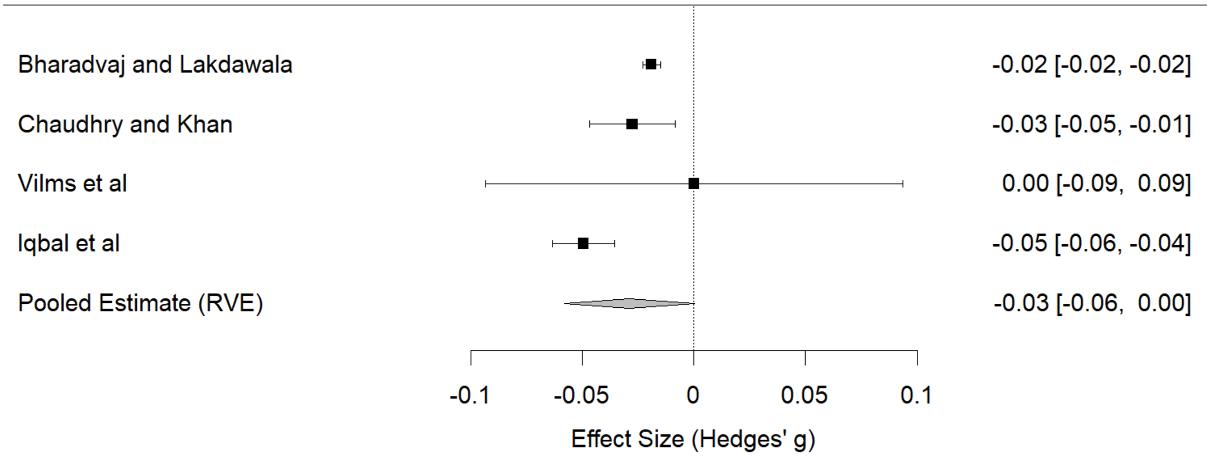

Figure S6. Forest plot of individual studies in healthcare expenditure and corresponding pooled RVE model.

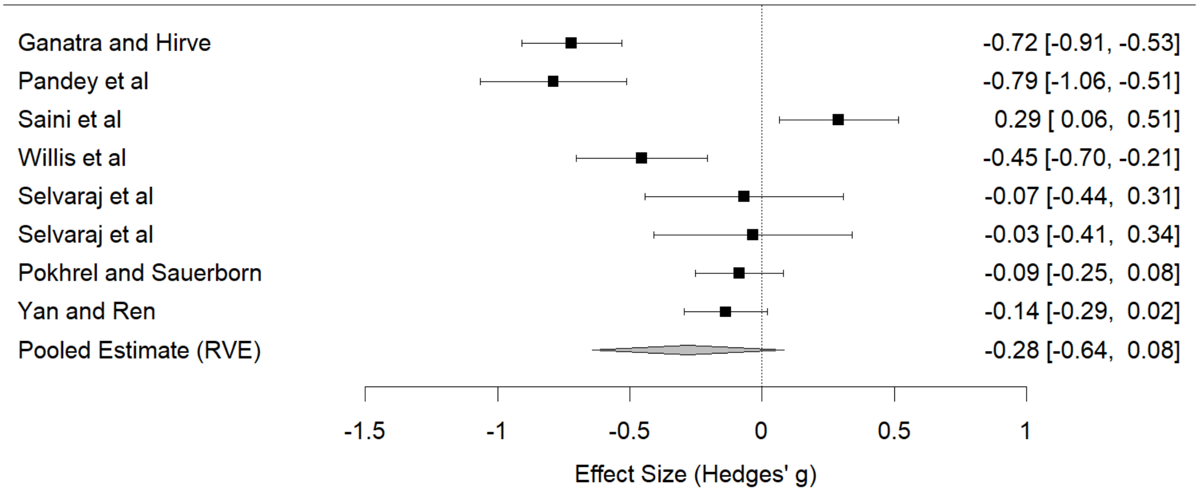

Figure S7 and S8. Publication bias assessment plots.

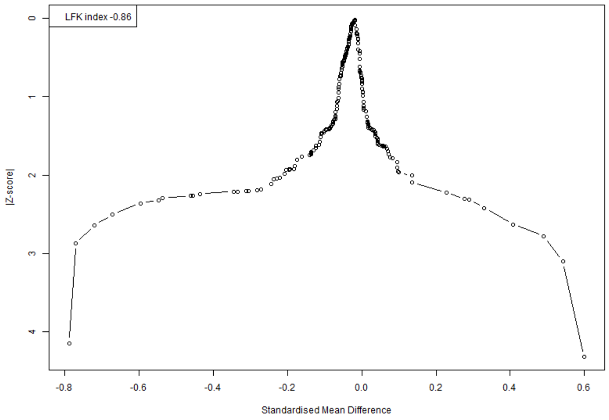

Note: Calculations are based on effect sizes from 52 studies included in the meta-analysis.

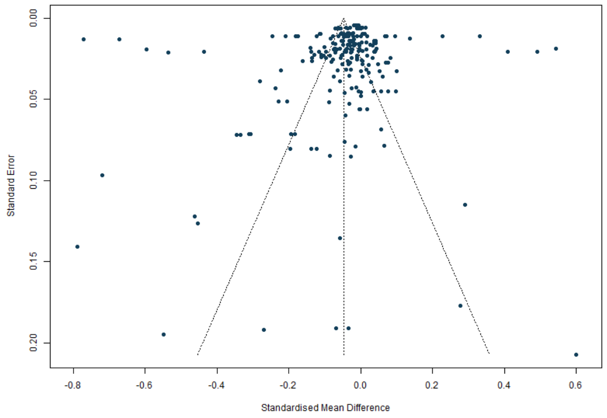

Note: Calculations are based on effect sizes from 52 studies included in the meta-analysis.

Figure S9. Number of papers over time.

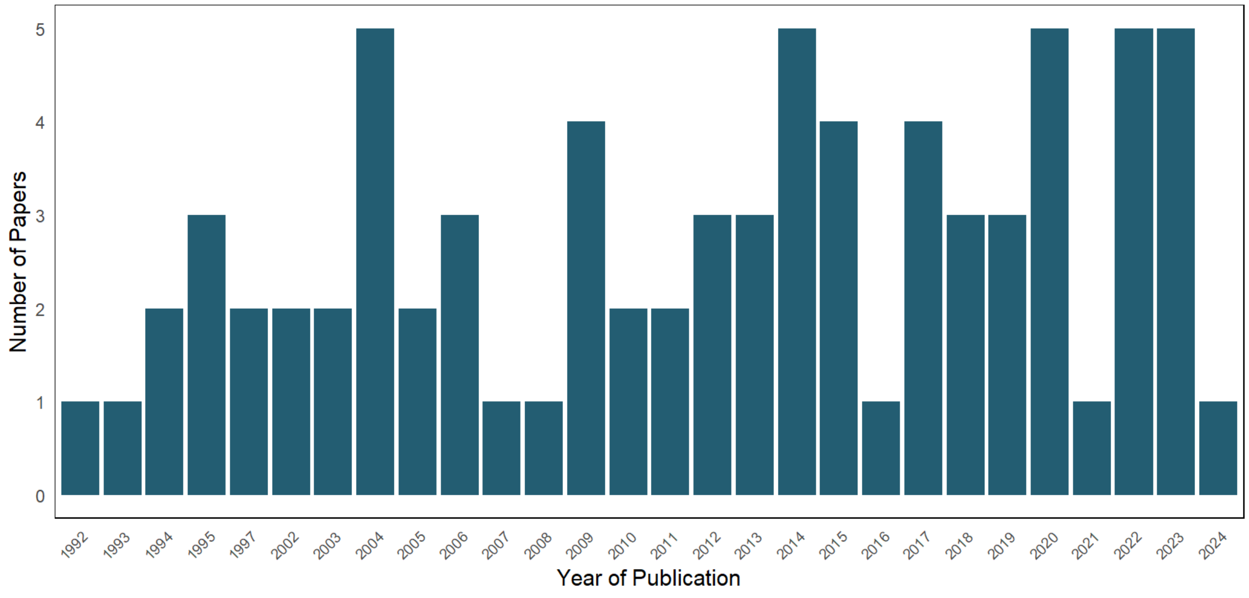

Note: Estimations are based on 78 included studies identified in this review.

Table S11. Quality assessment of included studies based on JBI quality appraisal tool

Study ID	Were the aims and objectives of the study clearly stated?	Were the criteria for inclusion in the sample clearly defined?	Were the study subjects described in detail?	Was the study setting described in detail?	Were the outcomes measured in a valid and reliable way?	Were the confounding factors identified?	Were strategies to deal with confounding factors stated?	Was appropriate statistical analysis used?	Were the limitations of the study discussed?	Quality
1	Yes	Yes	Yes	Yes	Unclear	Yes	NA	No	Yes	81%
2	Yes	Unclear	Yes	Yes	Yes	Yes	Unclear	Yes	Yes	89%
4	Yes	Yes	Yes	Unclear	Unclear	Yes	Yes	Yes	Yes	89%
5	Yes	Yes	Yes	Unclear	Unclear	Yes	Yes	Yes	Yes	89%
6	Yes	Yes	Yes	Unclear	Yes	Yes	No	Yes	No	72%
8	Yes	Unclear	No	Unclear	Yes	Yes	Yes	Yes	No	67%
9	Yes	Yes	Unclear	No	Unclear	Yes	Unclear	Yes	Unclear	67%
10	Yes	Yes	Yes	Yes	Unclear	Yes	Yes	Yes	Yes	94%
11	Yes	Yes	Yes	Yes	Yes	Yes	Yes	Yes	Yes	100%
12	Yes	No	Yes	Yes	Unclear	NA	NA	No	Unclear	57%
13	Yes	Yes	Yes	Yes	Yes	Yes	Unclear	Yes	Yes	94%
14	Yes	Yes	No	Unclear	No	Yes	No	Yes	Unclear	56%
15	Yes	Yes	Yes	Yes	Unclear	Yes	Yes	Yes	No	83%
16	Yes	Yes	Yes	Unclear	Yes	Unclear	No	Yes	Unclear	72%
17	Yes	Unclear	Yes	Yes	Yes	Yes	Unclear	Yes	Yes	89%
18	Yes	Yes	Yes	No	Yes	Yes	Unclear	Yes	No	72%
19	Yes	Yes	Yes	Unclear	Yes	Yes	Yes	Yes	Yes	94%

21	Yes	Unclear	Yes	Unclear	Yes	NA	NA	No	Unclear	64%
23	Yes	Unclear	Yes	Yes	Yes	Yes	Unclear	Yes	No	78%
24	Yes	Yes	Yes	Yes	Unclear	Yes	Yes	Yes	Unclear	89%
25	Yes	Yes	Yes	Unclear	Unclear	Yes	Unclear	Yes	Yes	83%
26	Yes	Unclear	Yes	Yes	Unclear	Yes	No	Yes	Yes	78%
28	Yes	Yes	Yes	Yes	Unclear	Yes	Unclear	Yes	No	78%
29	Yes	Unclear	Yes	Yes	Unclear	NA	NA	No	No	57%
30	Yes	Yes	Yes	Unclear	Yes	Yes	No	Yes	No	72%
31	Yes	Yes	Yes	Yes	Unclear	NA	NA	No	No	64%
32	Yes	Unclear	No	Yes	Yes	Unclear	No	Yes	Unclear	61%
33	Yes	Yes	Yes	Yes	Unclear	Yes	No	Yes	No	72%
34	Yes	Yes	Yes	Yes	Yes	Yes	No	Yes	No	78%
36	Yes	Yes	Yes	Yes	Yes	No	No	No	No	56%
37	Yes	Unclear	Yes	Unclear	Unclear	No	NA	No	No	44%
38	Yes	Yes	Yes	Unclear	Unclear	Yes	Yes	Yes	Yes	89%
39	Yes	Unclear	Yes	Yes	Unclear	Yes	Unclear	Yes	Yes	83%
40	Yes	Unclear	Yes	Yes	Yes	Yes	No	Yes	No	72%
41	Yes	Unclear	Yes	No	Yes	Unclear	No	Yes	Unclear	61%
42	Yes	No	Unclear	No	Unclear	NA	NA	No	No	29%
43	Yes	Unclear	Yes	No	Unclear	Unclear	No	Yes	No	50%
45	Yes	Yes	Yes	Unclear	Yes	Yes	No	Yes	Yes	83%
46	Yes	Yes	Yes	Unclear	Yes	Yes	No	Yes	Yes	83%
47	Yes	Yes	Yes	Unclear	Yes	NA	NA	No	No	64%

48	Yes	Yes	Yes	NA	Unclear	Yes	No	Yes	Yes	81%
51	Yes	Yes	Yes	Unclear	No	No	No	No	No	39%
52	Yes	Yes	Yes	No	Unclear	NA	NA	No	No	50%
53	Yes	Yes	Yes	Yes	Unclear	NA	NA	No	No	64%
54	Yes	Yes	Yes	No	Unclear	NA	NA	No	No	50%
55	Yes	Yes	Yes	Unclear	Yes	NA	NA	No	No	64%
56	Yes	Yes	Yes	No	Yes	Yes	Yes	Yes	Unclear	83%
58	Yes	Yes	Yes	Yes	Yes	Yes	No	Yes	Unclear	83%
59	Yes	Yes	Yes	No	Yes	Unclear	No	Yes	Unclear	67%
60	Yes	Unclear	Yes	No	Yes	Unclear	No	Yes	No	56%
61	Yes	Yes	Yes	Yes	Unclear	Yes	Yes	Yes	Yes	94%
62	Yes	Yes	Yes	No	Unclear	NA	NA	No	No	50%
63	Yes	Yes	Yes	Unclear	Unclear	Yes	Yes	Yes	Unclear	83%
65	Yes	Yes	Yes	No	Unclear	Yes	Unclear	Yes	No	67%
66	Yes	Yes	Yes	Yes	Unclear	Unclear	No	No	Yes	67%
67	Yes	Yes	Unclear	Yes	Yes	Yes	No	No	Yes	72%
68	Yes	Yes	Yes	Yes	Yes	No	No	No	Yes	67%
71	Yes	Yes	No	Yes	Unclear	No	No	No	Yes	50%
73	Yes	Yes	No	Yes	Unclear	Yes	Unclear	Yes	Unclear	72%
74	Unclear	Yes	Yes	Yes	Yes	Yes	Unclear	Yes	Yes	89%
75	Yes	Unclear	Yes	Yes	Yes	Yes	No	Yes	Yes	83%
76	Yes	Yes	Yes	Unclear	Yes	Yes	No	Yes	No	72%
79	Yes	Yes	Yes	Unclear	Yes	Yes	No	Yes	Yes	83%

80	Yes	Yes	Yes	No	Yes	Yes	No	Yes	Yes	78%
82	Yes	Unclear	Yes	Unclear	Unclear	Yes	No	Yes	Yes	72%
83	NA	NA	NA	NA	NA	NA	NA	NA	NA	-
84	Yes	Yes	Yes	Yes	Yes	NA	NA	No	Yes	86%
86	Unclear	Unclear	No	No	No	No	No	No	Yes	22%
87	Yes	Yes	Yes	Yes	Yes	Yes	No	Yes	Yes	89%
88	Yes	Yes	Yes	Yes	Yes	No	No	No	Yes	67%
89	Yes	Yes	Yes	Yes	Yes	No	No	No	Yes	67%
90	Yes	Yes	Yes	Yes	Yes	Yes	Yes	Yes	Yes	100%
91	Yes	Yes	Unclear	Yes	Unclear	Yes	No	Yes	No	67%
92	Yes	Yes	Yes	No	Yes	No	No	Yes	No	56%
93	Yes	Yes	No	Yes	Yes	Yes	Unclear	Yes	No	72%
94	Yes	Yes	Unclear	No	No	No	No	Unclear	No	33%
95	Yes	Yes	Yes	Yes	Yes	Yes	Yes	Yes	Yes	100%
96	Yes	Yes	Yes	Yes	Yes	Yes	Yes	Yes	Yes	100%

Note: NA= Not applicable
